# Role for a Novel Usher Protein Complex in Hair Cell Synaptic Maturation

**DOI:** 10.1371/journal.pone.0030573

**Published:** 2012-02-17

**Authors:** Marisa Zallocchi, Daniel T. Meehan, Duane Delimont, Joseph Rutledge, Michael Anne Gratton, John Flannery, Dominic Cosgrove

**Affiliations:** 1 Boys Town National Research Hospital, Omaha, Nebraska, United States of America; 2 Otolaryngology-Head, Neck Surgery, St Louis University, St Louis, Missouri, United States of America; 3 Helen Wills Neuroscience Institute, University of California, Berkeley, California, United States of America; 4 University of Nebraska Medical Center, Omaha, Nebraska, United States of America; Emory University, United States of America

## Abstract

The molecular mechanisms underlying hair cell synaptic maturation are not well understood. Cadherin-23 (CDH23), protocadherin-15 (PCDH15) and the very large G-protein coupled receptor 1 (VLGR1) have been implicated in the development of cochlear hair cell stereocilia, while clarin-1 has been suggested to also play a role in synaptogenesis. Mutations in CDH23, PCDH15, VLGR1 and clarin-1 cause Usher syndrome, characterized by congenital deafness, vestibular dysfunction and *retinitis pigmentosa*. Here we show developmental expression of these Usher proteins in afferent spiral ganglion neurons and hair cell synapses. We identify a novel synaptic Usher complex comprised of clarin-1 and specific isoforms of CDH23, PCDH15 and VLGR1. To establish the *in vivo* relevance of this complex, we performed morphological and quantitative analysis of the neuronal fibers and their synapses in the *Clrn1−/−* mouse, which was generated by incomplete deletion of the gene. These mice showed a delay in neuronal/synaptic maturation by both immunostaining and electron microscopy. Analysis of the ribbon synapses in *Ames waltzer^av3J^* mice also suggests a delay in hair cell synaptogenesis. Collectively, these results show that, in addition to the well documented role for Usher proteins in stereocilia development, Usher protein complexes comprised of specific protein isoforms likely function in synaptic maturation as well.

## Introduction

Inner hair cells (IHCs) are the cochlear mechano-electrical sensors that transduce sound waves into electrical currents [1], [2]. They are innervated by several synapses formed by type I afferent spiral ganglion neurons (SGNs), facing a synaptic ribbon surrounded by microvesicles, and lateral efferent fibers. Outer hair cells (OHCs) are the sound-evoked cochlear amplifiers and make synaptic contacts with type II afferent SGNs and efferent fibers [3]–[5]. While there is a transient developmental innervation of the mouse OHCs by type I afferent fibers, these are retracted before the onset of hearing when the type I afferent neurons specifically innervate the base of the IHCs [6]–[9].

Usher syndrome (USH) is a genetic neurosensory disorder characterized by congenital deafness, variable vestibular dysfunction and progressive *retinitis pigmentosa* [10], [11]. Among the 9 genes associated with USH, mutations in the genes encoding adhesion molecules, cadherin-23 (CDH23) and protocadherin-15 (PCDH15), the very large G protein-coupled receptor 1 (VLGR1) and clarin-1 are associated to USH1D, USH1F, USH2C and USH3A, respectively [12]–[15]. It has been suggested that these Usher proteins play roles in hair cell development, neurogenesis and synaptogenesis [16]–[19].

Most of the earlier work describing interactions between USH related proteins was based on *in vitro* studies using heterologous expression systems [20]–[22]. Our own work and that of others, demonstrate that some of these interactions occur *in vivo* [23], [24]. These data combined with immunolocalization studies and a splayed stereocilia phenotype shared by Usher mouse models support a widely accepted view that Usher protein interactions play a key role in stereocilia development and function [25]–[28].

There have been several reports demonstrating Usher protein expression at the synapses of hair cells and photoreceptors [16], [19], [22], [29], [30]. Based on these observations we explored the possible existence and potential functional relevance of a synaptic Usher complex(es) in cochlear hair cells. We observed pre- and post-synaptic expression of the Usher proteins in IHCs and OHCs during synaptic development and in mature IHCs. We demonstrate the existence of a synaptic complex comprised of clarin-1 and specific isoforms of CDH23, PCDH15 and VLGR1. We show a delay in cochlear hair cell synaptogenesis in the clarin-1 knockout mice (*Clrn1*−/−) as well as *Ames waltzer* mice (PCDH15 mutants). Combined, these data suggest that these Usher proteins function as a complex in early phases of synaptic maturation.

## Materials and Methods

### Animals

Wild-type (WT) mice (post-natal day 1 [P1] to P60) were in the 129Sv/J strain and obtained from Jackson Laboratories (BarHarbor, ME). P3 to P14 *Clrn1*−/− mice [31], P9 *Ames waltzer^av3J^* mice [32] and strain age-matched controls were on the C57BL/6J background. All mice were bred in-house. Experiments using mice were carried out under an approved IACUC protocol, and every effort was made to minimize pain and discomfort.

### Antibodies

**Figure 1:**
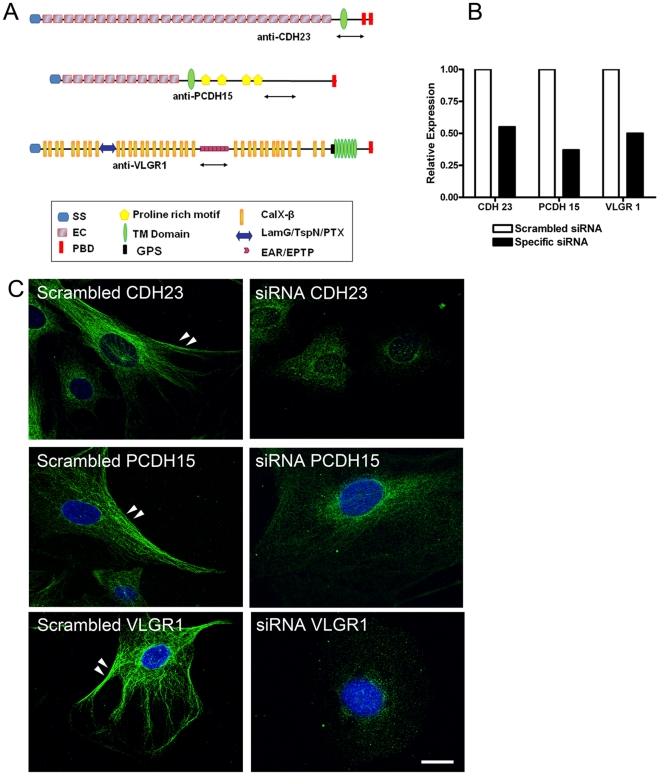
Isoform specificity by transient knockdown in differentiated UB/OC-1 cells. **Panel A:** Predicted structure and domains of full length CDH23, PCDH15 and VLGR1 with the regions comprising the peptide immunogens indicated as double–headed arrows. SS: signal sequence. EC: extracellular cadherin repeats. PBD: PDZ binding domain. TM: transmembrane domain. GPS: G-protein-couple receptor proteolytic site. CalX-β: Ca^2^+-binding domain of the Na+/Ca^2^+ exchanger molecule. LamG/TspN/PTX: laminin-G/thrombospondin-N/pentraxin homology domains. EAR/EPTP: epilepsy-associated repeat/epitempin repeat. Domains are not to scale. **Panels B–D:** Differentiated UB/OC-1 cells were transiently transfected with scrambled siRNA or siRNAs specific for mouse *Cdh23*, *Pcdh15* or *Vlgr1* transcripts. **Panel B:** Real time qRT-PCR. Histogram shows percentage of mRNA expression relative to scrambled. **Panel C:** Immunocytochemistry with anti-CDH23 (top), anti-PCDH15 (middle) and anti-VLGR1 (bottom). Scale bar: 10 µm. Arrowheads denote plasma membrane localization (there were no differences between WT and Scrambled cells, data not shown).

The rabbit polyclonal anti-clarin-1 was developed in our laboratory, described and qualified by siRNA knockdown, peptide competition assay and the use of knockout animals [19], [33]. Rabbit polyclonal anti-CDH23, anti-PCDH15 and anti-VLGR1 antibodies were also developed by our laboratory, described and qualified previously [24], [27], [34]. Fusion peptide constructs for CDH23, PCDH15 and VLGR1 were produced and expressed in *E.coli* as described in Zallocchi et al. [24] and antibodies were raised in rabbits under contract with Chemicon (Temecula, CA). Anti-CDH23 was raised against a peptide comprising amino acids 2954 to 3173, PCDH15 fusion peptide comprises amino acids 1490 to 1709 of the PCDH15 CD1 isoform [35] and VLGR1 antibody was raised against a peptide that corresponds to amino acids 3245–3421 (**Fig. 1A** and **Table S1**).

Other antibodies used in this work were mouse anti-myosin7A [36], mouse anti-RIBEYE (BD Biosciences, CA), mouse monoclonal anti-β-actin (Sigma, MO), mouse anti-SNAP25 (Abcam, MA), goat anti-PMCA2 (Santa Cruz, CA), rabbit anti-glutamate receptor 2/3 (GluR2/3) and chicken polyclonal anti-peripherin (Chemicon, CA).

### Immunofluorescence

#### Cochlea

Tissue was prepared as described by Zallocchi et al. [19] using 10% of fetal calf serum and 0.1% of Triton X-100 in PBS as blocking solution. Primary antibody dilutions were 1∶800 for anti-PCDH15, anti-CDH23 and anti-VLGR1, 1∶2,000 for anti-clarin-1, 1∶200 for anti-peripherin and 1∶100 for anti-RIBEYE and anti-myosin7A. Secondary antibodies were Alexa Fluor conjugated antibodies (Invitrogen, NY). Controls with pre-immune serum were done to test specificity of the antibodies.

#### Whole mount

For the study of the ribbon synapses, organs of Corti from wild type (WT), *Clrn1*−/− and *Ames waltzer^av3J^* mice were prepared according to Sendin et al. [37].

#### Hair cells

Hair cells isolation from P3 and P30 organs of Corti was done according to Legendre et al. [38] with some modifications. Briefly, microdissected organs of Corti were incubated in the presence of 0.5% papain (Sigma, MO) for 10 (P30) or 20 (P3) minutes at room temperature, followed by a gentle trituration to liberate the hair cells. Primary antibody dilutions were 1∶250 for all the Usher antibodies in fish gelatin blocking solution (2% fetal calf serum, 0.2% BSA, 0.3% fish gelatin in PBS).

#### UB/OC-1 cells

Differentiated UB/OC-1 (University of Bristol/organ of Corti-1) cells were grown and processed for confocal microscopy as described by Zallocchi et al. [19]. Primary antibody dilutions were 1∶500 for anti-PCDH15 and 1∶1,000 for anti-VLGR1 and anti-CDH23 in fish gelatin blocking solution.

### Neuronal tracer application

WT and *Clrn1*−/− type I afferent fibers were labeled as described elsewhere [19], [39], [40]. Cross-sections were either immunostained for the specific Usher antibodies (dilution 1∶250) or for F-actin with phalloidin-Alexa Fluor-488.

### Confocal microscopy

Slides were coverslipped using Vectashield mounting medium containing DAPI to counter-stain the nuclei (Vector Lab. CA) and confocal images captured using a Zeiss AxioPlan 2IF MOT microscope interfaced with a LSM510 META confocal imaging system. We used 20X NA: 0.5, 63X NA: 1.4 and 40X NA: 1.3 oil objectives. To produce three-dimensional reconstructions of a specimen (whole mounts), a *z-*axis stack of two-dimensional images was taken with a step size of 0.5 µm. Final figures were assembled using Adobe Photoshop and Illustrator software (Adobe Systems, CA).

### Electron microscopy

P9 mouse cochleae were fixed with 4% PFA/2% glutaraldehyde in 0.2 M phosphate buffer (pH 7.4), post-fixed with 1% Osmium tetroxide and then decalcified in 0.12 mM EDTA. After dehydration and infiltration, cochleae were embedded in resin (Embed 812), bisected through the modiolus and re-embedded. Mid-modiolar thin sections (70 nm) were placed on formvar-coated copper slot grids and counterstained with Uranyl Acetate and Lead Citrate. Sections were viewed on a Hitachi H-7500 transmission electron microscope. Digitized images were obtained and archived with a CCD camera and AMT 12-HR software (version 5.4.2.239). Final figures were assembled using Adobe Photoshop and Illustrator software (Adobe Systems, CA).

### Cell culture

Cultivation of UB/OC-1 was conducted as previously reported [19], [41], [42]. Differentiated UB/OC-1 cells were used as a source of protein or dissociated and plated onto poly-L-lysine coated microscope slides (VWR, IL) for immunofluorescence analysis (described above).

### Transient siRNA knockdown

UB/OC-1 cells were electroporated in the presence of scrambled siRNA or siRNAs specific for the different Usher proteins, as described by Zallocchi et al. [19]. Scrambled and the specific siRNAs were directed to the sequences used to derive the peptide immunogens for each Usher transcript, and were designed by Applied Biosystems (Foster City, CA.). Sense and antisense sequences of the corresponding siRNAs are as follows (see also **Table S1**): CDH23: GAACUUUGCGCAGACAGAAtt/UUCUGUCUGCGCAAAGUUCac. PCDH15: GAACAGAUUUUGAAGAGCUtt/AGCUCUUCAAAAUCUGUUCgt. VLGR1: AGUCAAGACUAUUUCAUCAtt/UGAUGAAAUAGUCUUGACUgt. Knockdown cells were qualified by real-time quantitative RT-PCR using SYBR® Green PCR Master Mix (Applied Biosystems, CA), according to the manufacturer protocol, normalized with β-actin transcript abundance, and compared with the scrambled siRNA. Real time PCR primers (**Table S1**): CDH23: AGAAAGCTAAAGGCCATTGTG/GTCTGCGATCTCACTCAGGTT. PCDH15: GAGGGTGTGGAATCAGTCTG/GAACGTTGCTGCTACCTCTC. VLGR1: ATGAGGGGAATGGATGTCGTC/ATATGGGGAAACGCCAATATTAAAGG. A conventional PCR with the real time primers was run using WT cDNA from UB/OC-1 cells as template and the PCR products were sequence verified.

Knockdown cells were processed for immunofluorescence and western blot analysis. Specific bands were quantified using the software ImageJ 1.43 from the National Institutes of Health, USA, rsb.info.nih.gov/ij.

RACE reactions. To amplify the different alternative spliced isoforms of VLGR1, specific primers were designed within the immunogen region (exon 48) and used to perform rapid amplification of cDNA ends (RACE). RNA was isolated from P3 inner ear, extracted using TRIzol and DNAse treated (Invitrogen, NY). Three micrograms of total RNA were used for 5′ and 3′ RACE, performed with the SMARTer™ RACE Amplification kit (Clontech, CA). Running conditions were set as to obtain products no longer than 5-kb and according to the manufacturer instructions. The primer sequences were GGTGAAATGCCGTACTTGACAGCTTCCTG and CAGGAAGCTGTCAAGTACGGCATTTCACC for the 5′ and 3′ RACE, respectively. The 4886-bp (Vlgr1 f.1, GenBank Acc. Number: JQ013292) and 780-bp (Vlgr1 g.1, GenBank Acc. Number: JQ013291) products of the 5′ RACE and the 800-bp (Vlgr1 f.2, GenBank Acc. Number: JK693834) product of the 3′ RACE were purified, cloned into the TOPO TA vector cloning kit (Invitrogen, NY) and sequenced by the University of Nebraska Medical Center DNA-Sequencing Core Facility.

### Co-immunoprecipitation (co-IP) studies

Differentiated UB/OC-1 cells were used for co-IP studies. In a standard experiment eight 150 cm^2^ dishes were used and cells were processed for the isolation of membranes as described by Le-Niculescu et al. [43].

Sixty microliters of a 50% slurry suspension of protein A-sepharose beads (Sigma, MO) were incubated overnight at 4°C with 10 µl of the specific Usher antibodies, divided into two equal aliquots and incubated overnight in the presence of UB/OC-1 membrane fraction (specific Usher antibody+M) or buffer (specific Usher antibody+B, *control 1*). The membrane fraction was also incubated overnight in the presence of normal rabbit serum coated beads (NRS+M, *control 2*) or un-coated beads (NO AB+M, *control 3*). After several washes, co-IPs were resuspended in sample buffer and analyzed by western blotting.

### Lysates and synaptosomal preparations

#### Lysates

Organs of Corti, neuroretinas or differentiated UB/OC-1 cells were homogenized in RIPA buffer (150 mM NaCl, 50 mM Tris, 1 mM EDTA, 1% NP-40, 0.5% sodium deoxycholate, 0.1% SDS, pH 7.4) containing protease inhibitors, and ten to thirty micrograms of protein (Bio-Rad CA. 500–0006) [44] were analyzed by western blotting. In the case of UB/OC-1 knockdown cells, due to the limited amount of material after the electroporation only three to five micrograms were analyzed. **Crude synaptosomes** from neuroretina were isolated as described by Reiners et al. [45], with adaptation to the inner ear. Briefly, 16 organs of Corti from P3 WT mice were homogenized in 200 µl of extraction buffer (0.32 M sucrose, 4 mM Hepes [pH 7.4], and protease inhibitors) and the post-nuclear supernatant was centrifuged at 9,200×*g* for 15 min. The new supernatant (S1) was kept for western blot analysis while the pellet, containing the synaptosomes, was resuspended and centrifuged at 10,800×*g* for 15 min. The new pellet containing the crude synaptosomal fraction (P2) was analyzed by western-blot.

### Western blot

Western blot analysis was conducted as previously described by Zallocchi et al. [24]. Secondary antibody was replaced by HRP-conjugated protein A (GE Healthcare Life Sciences, NJ) or HRP-conjugated protein-G (Chemicon, CA) dilution 1∶20,000 to avoid or decreased cross-reactivity with the IgGs present in the tissue and co-IPs. Primary antibody dilutions: anti-VLGR1, anti-CDH23 and anti-PCDH15 2 µg/ml; anti-clarin-1 0.5 µg/ml, anti-RIBEYE and anti-SNAP25 1∶500, anti-PMCA2 1∶200 and anti-β-actin 1∶2,000. For antibody qualification the specific antibodies were pre-adsorbed with the immobilized peptide immunogen overnight at 4°C with rocking. The flow through (unbound) and the elute (affinity purified antibodies) were use for western-blot analysis of P3 inner ear and neuroretina.

## Results

### Antibody qualification

**Figure 2:**
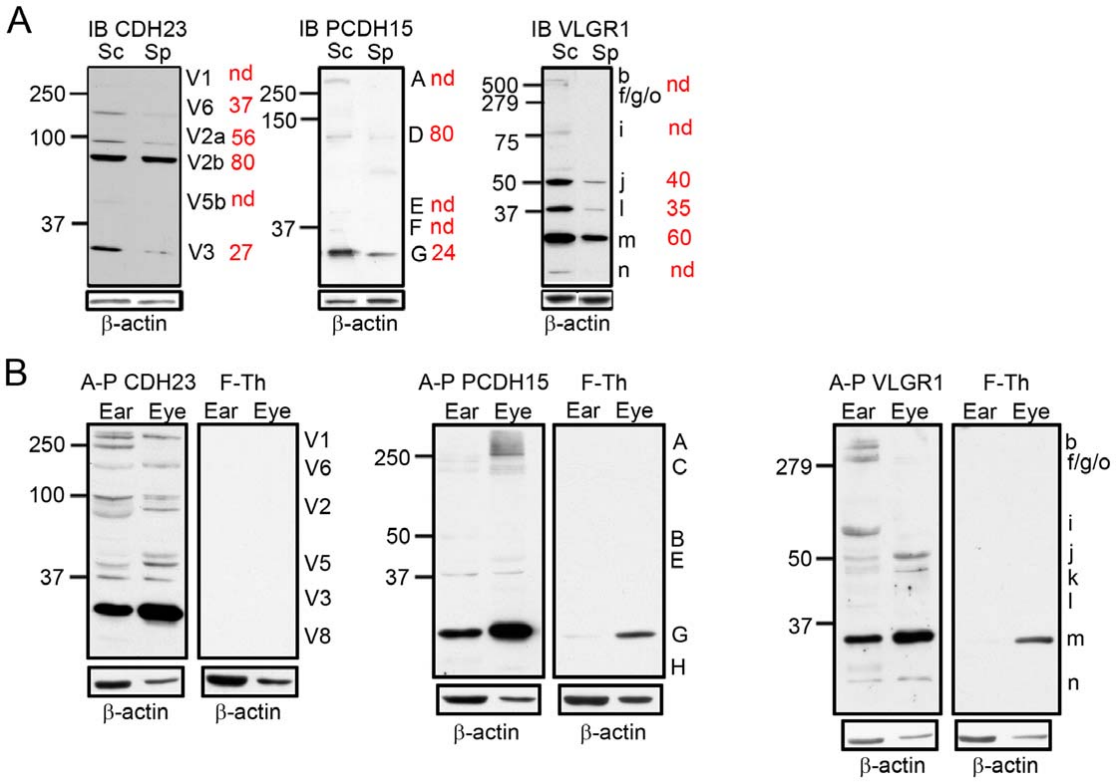
Isoform specificity by transient knockdown and antibody pre-adsorption. **Panel A:** Western blot analysis showing different degrees of knockdown for each isoform of the three different Usher proteins. Blots were stripped and re-probed for β-actin as a loading control. Red numbers are the percentage of expression relative to the scrambled and after normalization with β-actin. Isoforms were named according to previously establish nomenclature systems. Molecular protein standards are indicated to the left of each membrane. Sc: scrambled; Sp: specific siRNA, nd: no-detectable. **Panel B:** Western blot analysis of whole P3 inner ear (EAR) and neuroretina (EYE) in the presence of the affinity purify antibody (A–P) or the pre-adsorbed antibody (F-Th) by the corresponding peptide immunogen. Membranes were stripped and immunoblotted for β-actin as loading control. For CDH23, V1 corresponds to V1a and V1b, V2 corresponds to V2a and V2b, V3 corresponds to V3a and V3b and V5 corresponds to V5a and V5b.

To establish the specificity of the isoforms recognized by each of these antibodies given the lack of true knockout mouse models [29], [46]–[49], we employed two different approaches: specific knock down in UB/OC-1 cells (**Fig. 1** and **Fig. 2**) and pre-adsorption of the antibody with the peptide immunogen followed by western blot analysis **(** **Fig. 2**). We transiently knocked down each Usher transcript in differentiated UB/OC-1 cells with siRNAs targeted to a region within the immunogen sequence (**Fig. 1A**, double-headed arrows and **Table S1**). These cells were derived from a population of non-sensory epithelial cells in the greater epithelial ridge that, under differentiating conditions, adopt a hair-cell like phenotype [41], [42], and endogenously express all the known Usher proteins [19,41; this work and unpublished data]. The knockdown cells for CDH23, PCDH15 and VLGR1 were qualified by real-time quantitative RT-PCR with primers directed to the regions comprising the peptide immunogens (isoform specific primers) (**Fig. 1B** and **Table S1**) and by immunofluorescence analysis (**Fig. 1C**), and compared with cells transfected with scrambled siRNA (controls). Western blot analysis of these knockdowns shows a reduction of 20% to 100% for the different isoforms within a given Usher protein (**Fig. 2A**), confirming the antibody specificity for the various isoforms discussed in this work. Anti-CDH23 was raised against part of the extracellular domain, transmembrane domain and part of the cytoplasmic domain of the mouse CDH23 protein, including the amino acid sequence encoded by the alternative spliced exon 68 [28] (**Table S1** and **Fig. S1**). Any alternative spliced protein isoform containing part of or the entire region comprising the immunogen will be recognized by our antibody preparation. By using siRNAs targeted to this region (**Table S1**), we were able to knockdown most of these alternative spliced isoforms, including the full length (V1) and “synaptic” (V3) isoforms [28], [29]. In the case of PCDH15, the antibody was raised against part of the cytoplasmic domain (CD1 domain) [35], [50] of the mouse PCDH15 encoded by exon 35, which itself can be incompletely spliced out [51] (**Table S1** and **Fig. S1**). We detected and knocked down 5 different PCDH15 isoforms from UB/OC-1 lysates (**Fig. 2A**). Most of these PCDH15 isoforms have already been described at the transcript and/or protein level [12], [22], [23], [35], [51], [52]. Finally, the VLGR1 antibody was raised against a peptide comprising the mouse EAR/EPTP (epilepsy-associated repeat/epitempin repeat) domain (**Fig. 1A** and **Table S1**). The anti-VLGR1 antibody recognizes all the alternative spliced isoforms containing part of or the entire EAR/EPTP domain (**Fig. S1**). Using specific siRNAs directed to a small region of this domain we were able to knock down 7 different VLGR1 isoforms (**Fig. 2A**). For all the three Usher proteins we were able to knock down the isoforms relevant to this work (**Fig.** **2A** and additional Figures): CDH23 isoforms V1, V2, V3, V5b and V6, PCDH15 isoforms A (or CD1-1) [35], [53] and G and VLGR1 isoforms “b”, “f/g/o”, “i”, “j” and “m”.

Similar results were obtained when the antibodies were pre-adsorbed with the peptide immunogen followed by western blot analysis in P3 inner ear and neuroretina (**Fig. 2B**). Using the unbound suspension (F-Th) as probe following pre-adsorption of the antibodies shows a decreased or complete disappearance of the bands for the specific Usher proteins compared with the affinity purify antibodies (A–P), demonstrating that these bands are specifically recognized by our antibody preparations. In some cases we see a band in blots probed with the unbound fraction for PCDH15 and VLGR1 immunoblots. This is likely due to an incomplete adsorption of the antibody by the peptide immunogen as the band intensities are significantly decreased relative to the specific immunoblots.

### Spatiotemporal localization of CDH23, PCDH15 and VLGR1 proteins in mouse cochlea and hair cells

**Figure 3:**
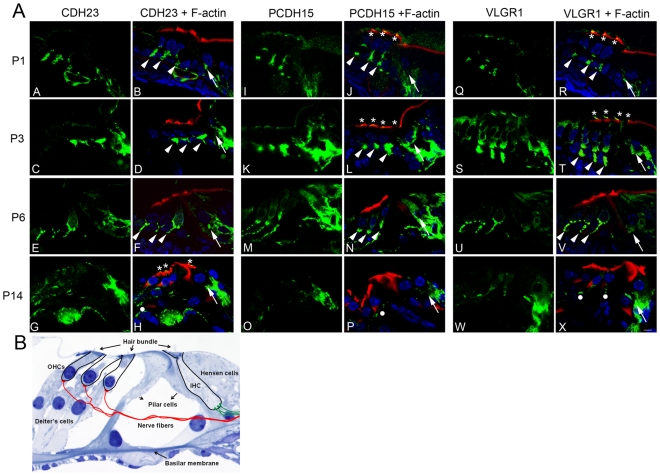
Developmental expression of Usher proteins in mouse cochleae. **Panel A:** Cochlea cross-sections immunostained with anti-CDH23 (**A–H**), anti-PCDH15 (**I–P**) and anti-VLGR1 (**Q–X**) (green) and counterstained with phalloidin for F-actin (red). P1 (**A, B, I, J, Q, R**); P3 (**C, D, K, L, S, T**); P6 (**E, F, M, N, U, V**) and P14 cochleae (**G, H, O, P, W, X**). Synaptic staining at the base of IHCs and OHCs is denoted by arrows and arrowheads, respectively. Asterisks: apical staining. White dots: weak immunostaining at the neuronal fibers. Scale bar: 5 µm. **Panel B:** Toluidine blue staining of P21 mammalian cochlea, for reference, showing the two different classes of hair cells and the innervation at their base. Magnification: 100×.

**Figure 4:**
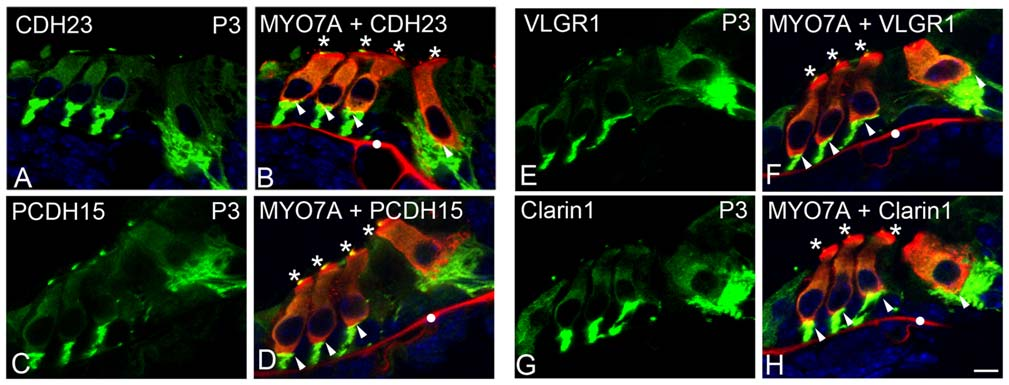
Basal expression of CDH23, PCDH15, VLGR1 and clarin-1 in P3 cochleae. Cochlea cross-sections were dual immunostained with the Usher proteins (green) and myosin7A (red). CDH23 (**A–B**); PCDH15 (**C–D**); VLGR1 (**E–F**) and clarin-1 (**G–H**). Arrowheads: basal pre-synaptic co-localization. Asterisks: apical co-localization. White dot: non-specific staining. Scale bar: 5 µm.

The expression of CDH23, PCDH15 and VLGR1 was analyzed in WT cochlea before (P1–P6) and after the onset of hearing (P14) [37] by dual immunofluorescence confocal microscopy. All sections were counterstained with phalloidin to identify hair cells by the presence of actin-rich stereocilia at the apical pole of the cells (**Fig. 3**). There is a specific spatiotemporal pattern of expression for these Usher proteins in cochlear hair cells and neuronal terminals between P1 to P14. As was reported by others, we observe expression of these proteins in the stereocilia of cochlear hair cells [27], [28], [32], [35], [47], [52]. Our antibody preparations detect apical expression of CDH23 at P3 and P14 (**Fig. 3G–H** and **Fig. 4**), and of PCDH15 and VLGR1 from P1 to P3 (**Fig. 3I–L, Q–T** and **Fig. 4**). There is also strong immunostaining at the base of both inner and outer hair cells, mainly post-synaptic, from P1 to P6 for these Usher proteins. At P14 the expression persists at the base of the IHCs (**Fig. 3**, arrows) and along the neuronal fibers that contact the OHCs (**Fig. 3**, white dots) but is mostly absent from the base of the OHCs. Basal staining for CDH23 and VLGR1 has previously been described by others [**29]**, [30], [49****]. No immunoreactivity was observed when sections where incubated with pre-immune serum and the secondary fluorochrome-labeled antibody, demonstrating the specificity of the staining (data not shown).

These results show a developmentally regulated pattern of expression at the hair cell basal pole and neuronal fibers for CDH23, PCDH15 and VLGR1 similar to the one previously reported for clarin-1 [16], [19] and coincident with the developmental window for type I afferent remodeling [6], [37]; [40].

**Figure 5:**
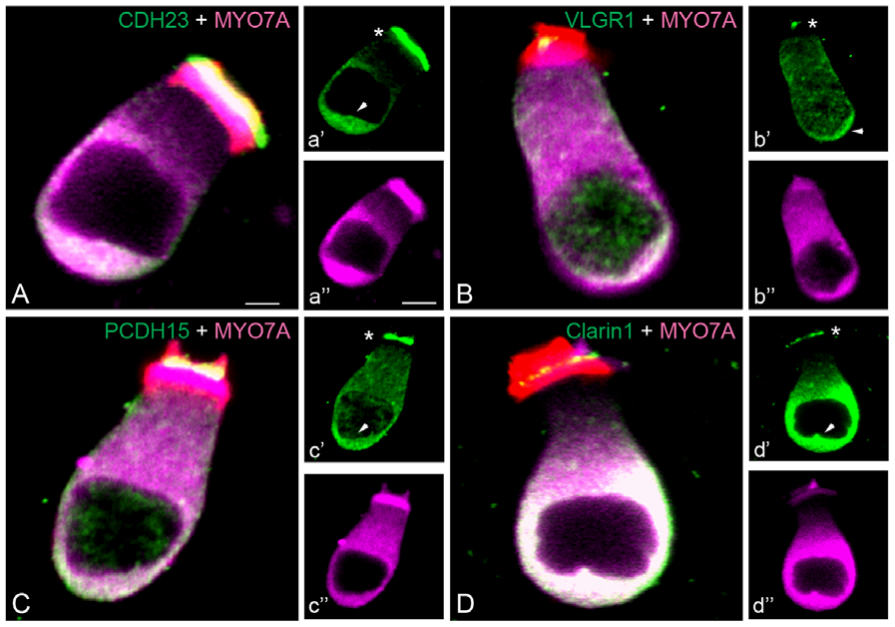
Usher proteins are present at the apical and basal aspects of P3 hair cells. Isolated hair cells were immunostained for the Usher proteins (green) and myosin7A (magenta) and counter-stained with phalloidin (red). Arrowheads: Usher staining at the base of the hair cells. Asterisks: apical staining and co-localization with phalloidin. **A a′, a″**: CDH23. **B, b′, b″:** VLGR1, **C, c′, c″:** PCDH15, **D, d′, d″:** clarin-1. Scale bar: **A–D:** 2 µm; **a′–d″:** 4 µm.

The basal expression of the Usher proteins, including clarin-1, was confirmed in P3 mouse cochleae by dual immunolabelling with the hair cell marker myosin7A [54] and in isolated hair cells. **Figure 4** shows co-localization at the base of both OHCs and IHCs between the Usher proteins and myosin7A (arrowheads) and at the stereocilia (asterisks). Apical and basal immunostaining were also observed for the four Usher proteins in isolated hair cells (green), with the basal staining enclosing the whole basolateral membrane (**Fig. 5**, arrowheads). It is worth mention that in our previous work [19] we were able to detect apical clarin-1 immunostaining at P0 only. The fact that now we observe apical expression at P3 (**Figs. 4** and **5**) and at P30 (**Fig. S2**) is likely methodological and due to changes in blocking solutions, the fixation, and in the case of the isolated hair cells the digestion treatment which reveals masked epitopes.

The pre-synaptic marker RIBEYE was also used to determine sub-cellular localization of the Usher proteins (**Fig. S3** and insets). Single plane confocal images show that CDH23, PCDH15, VLGR1 and clarin-1 not only co-localize with RIBEYE (arrowheads) that at P3 is expressed by both type of hair cells [6], [7], [9] but also extend their expression beyond the ribbon along the basolateral aspect of the hair cells. Particularly, in the case of IHCs there is a diffuse immunostaining for the Usher proteins along the cell body.

### CDH23, PCDH15, VLGR1 and clarin-1 are expressed by auditory nerve fibers (afferent fibers)

**Figure 6:**
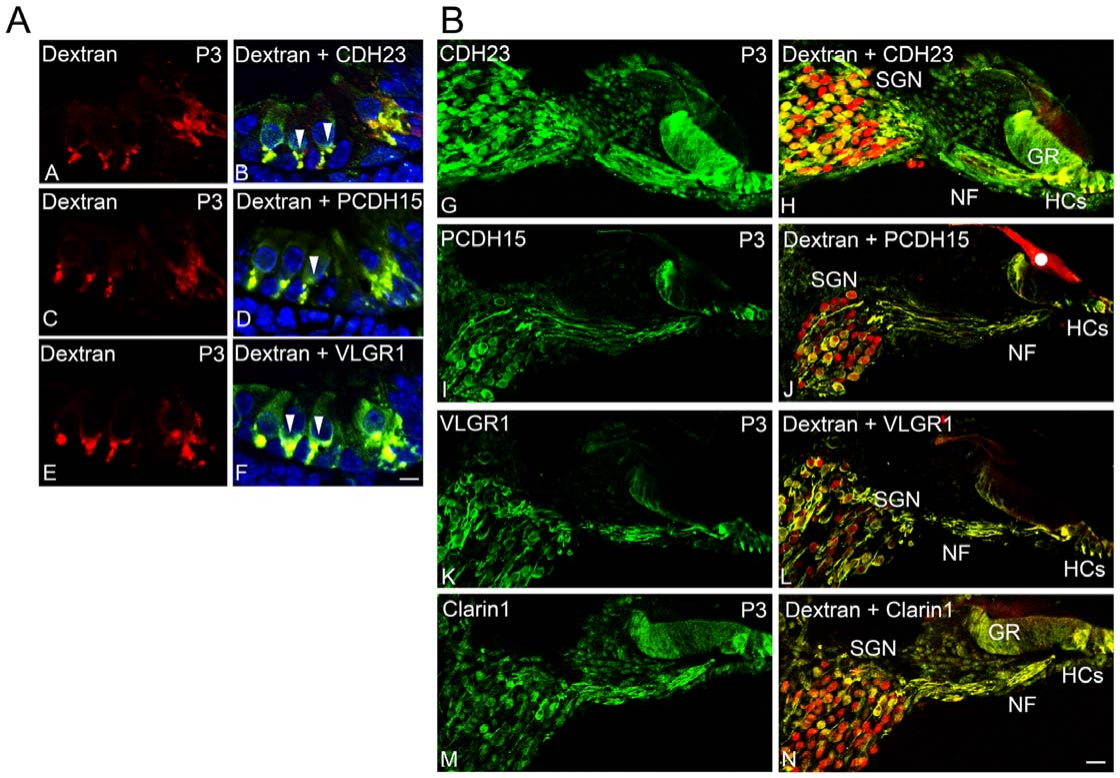
Usher protein expression by type I afferent synapses and corresponding SGNs. **Panel A:** P3 type I afferent fibers were labeled with a neuronal tracer (Texas Red conjugated-dextran) (**A–F**) and immunostained with anti-CDH23 (**B**), anti-PCDH15 (**D**) and anti-VLGR1 (**F**) (green). Arrowheads: pre-synaptic expression, indicated by the absence of co-localization with the neuronal tracer. Scale bar: 5 µm. **Panel B:** P3 cochlea cross-sections showing expression of the Usher proteins (green, **G–N**) in the spiral ganglion neurons (SGN), neuronal fibers (NF) and hair cells (HCs) and their co-localization with type I afferent neurons (red, **H, J, L, N**). GR: greater epithelial ridge cells. White dot: non-specific staining in the tectorial membrane. Scale bar: 20 µm.

The final steps in synaptic remodeling occur post-natally in mice and rats, where the type I afferent fibers retract from the base of the OHCs just prior to the onset of hearing [37], [40], [55]. Because the spatiotemporal pattern of the Usher proteins coincides with this synaptic remodeling event, we analyzed the origin of the Usher-positive neuronal fibers in P3 mouse cochleae. Type I afferent fibers were labeled with a fluorochrome-conjugated dextran, the cochleae were then cryosectioned and immunolabeled for the different Usher proteins [19], [39]; [40]. **Figure 6** shows co-localization between the Usher proteins and the dextran staining, demonstrating their expression by type I afferent terminal fibers (**Fig. 6** **, panel A**). Similar results were previously observed for clarin-1 [19]. The immunostaining with CDH23, PCDH15 and VLGR1 antibodies also shows pre-synaptic expression of the corresponding proteins, suggested by positive immunoreactivity at the base of the hair cells but absence of fluorochrome-conjugated dextran staining (**Fig. 6A**, arrowheads). The Usher proteins are not only present at the neuronal terminals and synaptic contacts but also in type I afferent fibers and SGNs (**Fig. 6** **, panel B**). The dual staining shows that only a sub-population of type I afferent neurons (yellow) expresses the Usher proteins and that these auditory nerve fibers are the ones that make synaptic contacts with the sensory epithelia. The greater epithelial ridge (GR) was also immunopositive for the Usher proteins, especially for CDH23.

**Figure 7:**
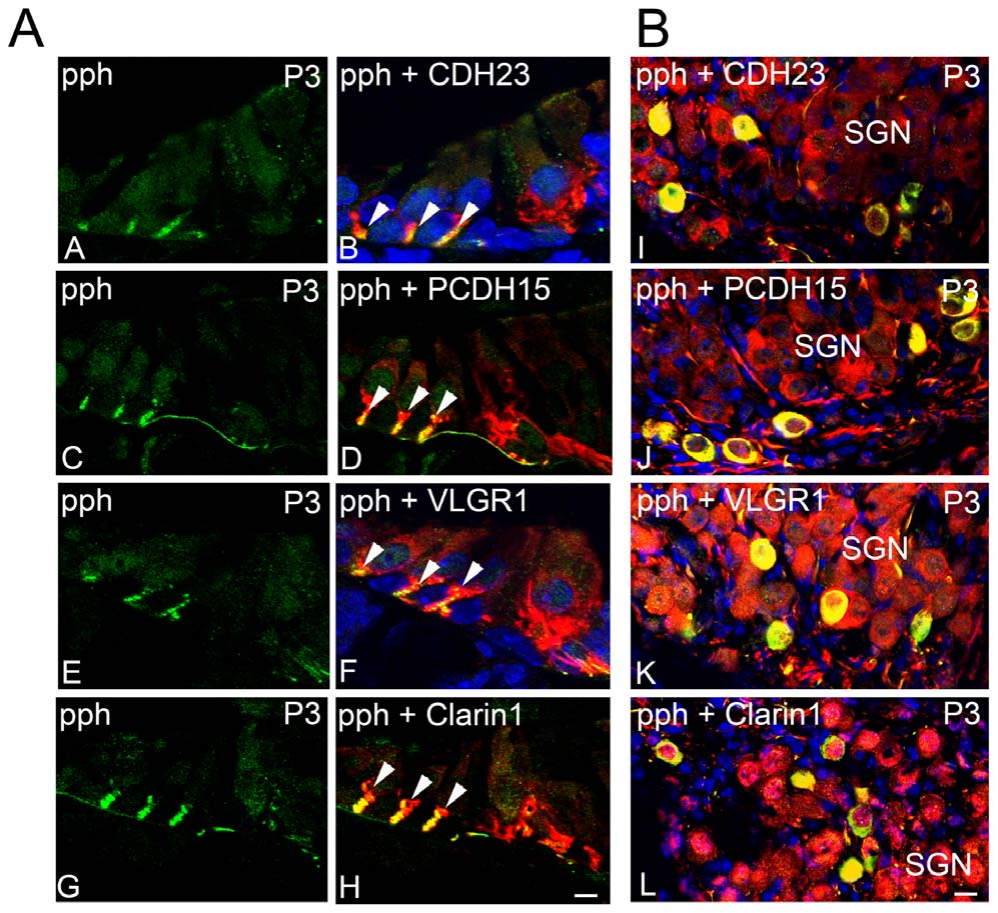
Usher protein expression by type II afferent neurons and SGN cell bodies. **Panel A:** P3 cochlea cross-sections dual-immunostained for peripherin (pph, green; **A–H**) and CDH23 (**B**), PCDH15 (**D**), VLGR1 (**F**) or clarin-1 (**H**) (red). Arrowheads: co-localization at the neuronal terminals. Scale bar: 5 µm. **Panel B:** P3 SGN cross-sections dual-immunostained with peripherin (green) and CDH23 (**I**), PCDH15 (**J**); VLGR1 (**K**) or clarin-1 (**L**) (red). Scale bar: 6 µm.

Because at this time point (P3) there is also type II afferent innervation at the base of the OHCs [40], we analyzed the possible expression of the Usher proteins by dual immunofluorescence with anti-peripherin, a marker for type II auditory nerve fibers [40]; [56]. **Figure 7** shows that CDH23, PCDH15, VLGR1 and clarin-1 are also expressed by type II afferent fibers and SGNs during early stages in synaptic cochlear development. In this case all the type II SGNs are positive for the Usher protein immunostaining (i.e. co-localization with peripherin, **Fig. 7** **, panel B**).

**Figure 8:**
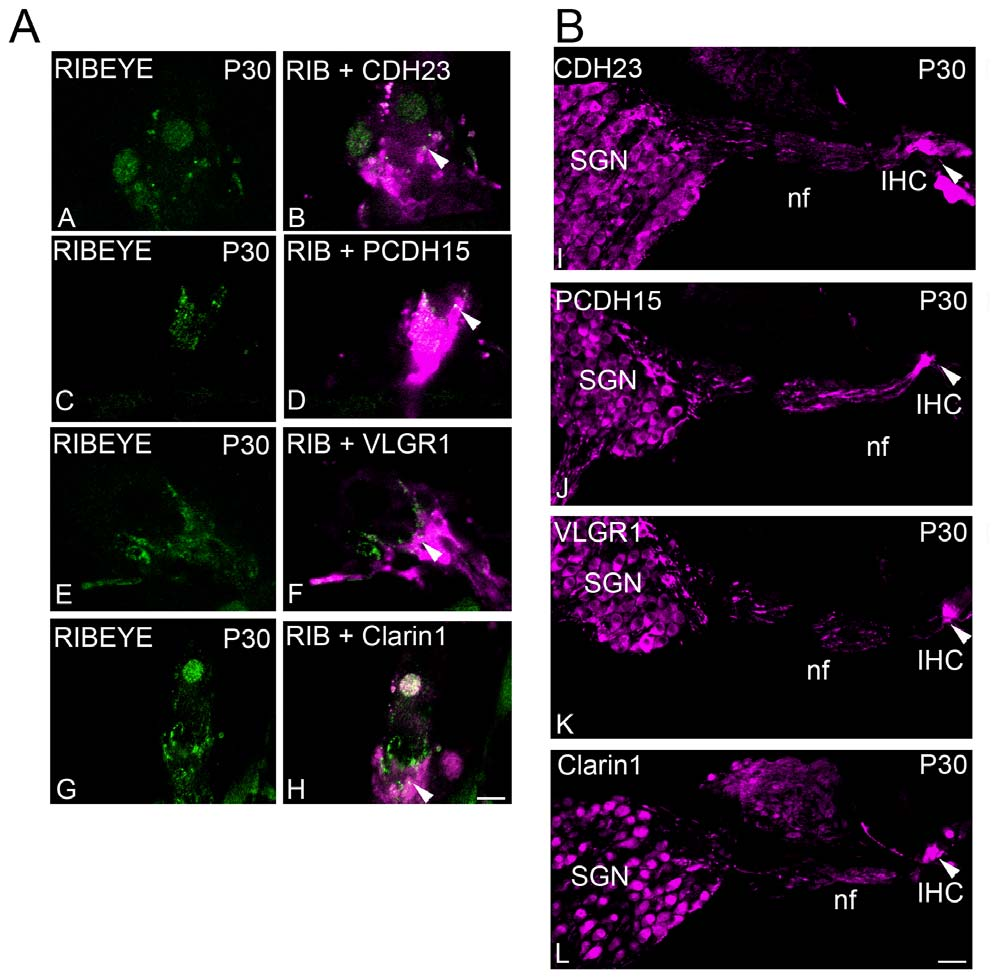
Expression of the Usher proteins in mature type I afferent neurons. **Panel A:** Single plane immunostaining of P30 IHCs with RIBEYE (**A–H**, green) and CDH23 (**B**), PCDH15 (**D**), VLGR1 (**F**) or clarin-1 (**H**) (magenta). Arrowheads denote co-localization. Scale bar: 6 µm. **Panel B:** P30 cross-sections immunostained for CDH23 (**I**), PCDH15 (**J**), VLGR1 (**K**) or clarin-1 (**L**), showing expression of the Usher proteins in the SGNs, type I afferent neuronal fibers (nf) and type I afferent terminals (arrowheads) that synapse the IHCs. Scale bar: 20 µm.

Expression of CDH23, PCDH15, VLGR1 and clarin-1 persists at the base of IHCs in adult mouse cochlea (**Fig. 8A**). This expression is post-synaptic in type I afferent SGNs and their neuronal fibers, that at P30 exclusively contact the base of the IHCs (**Fig. 8** **, panel B**, arrowheads). Very little co-localization, if any, is observed between the Usher proteins and RIBEYE (**Fig. 8A** arrowheads), suggesting that the basal isoforms are being down-regulated in the hair cells. Immunostaining of isolated hair cells from P30 cochleae (**Fig. S2**) and cochlea cross-sections from P60 animals (**Fig. S4**) confirm the absence of the basal immunostaining in mature hair cells. There is weak basal immunostaining for PCDH15 and a very strong immunostaining for the four Usher proteins at the stereocilia (**Fig. S2**). It is worth noting that we observe strong immunostaining in the apical region of the stereocilia with our VLGR1 antibody in isolated P30 hair cells (**Fig. S2**). This is not consistent with the transient “ankle link” staining described previously [27], and likely represents an isoform containing the EAR/EPTP domain but not the carboxyl-terminal domain of the protein, since the earlier studies utilized antibodies raised against the carboxy-terminal domain of VLGR1.

### Clarin-1 and specific isoforms of CDH23, PCDH15 and VLGR1 form protein complexes at hair cell synapses

**Figure 9:**
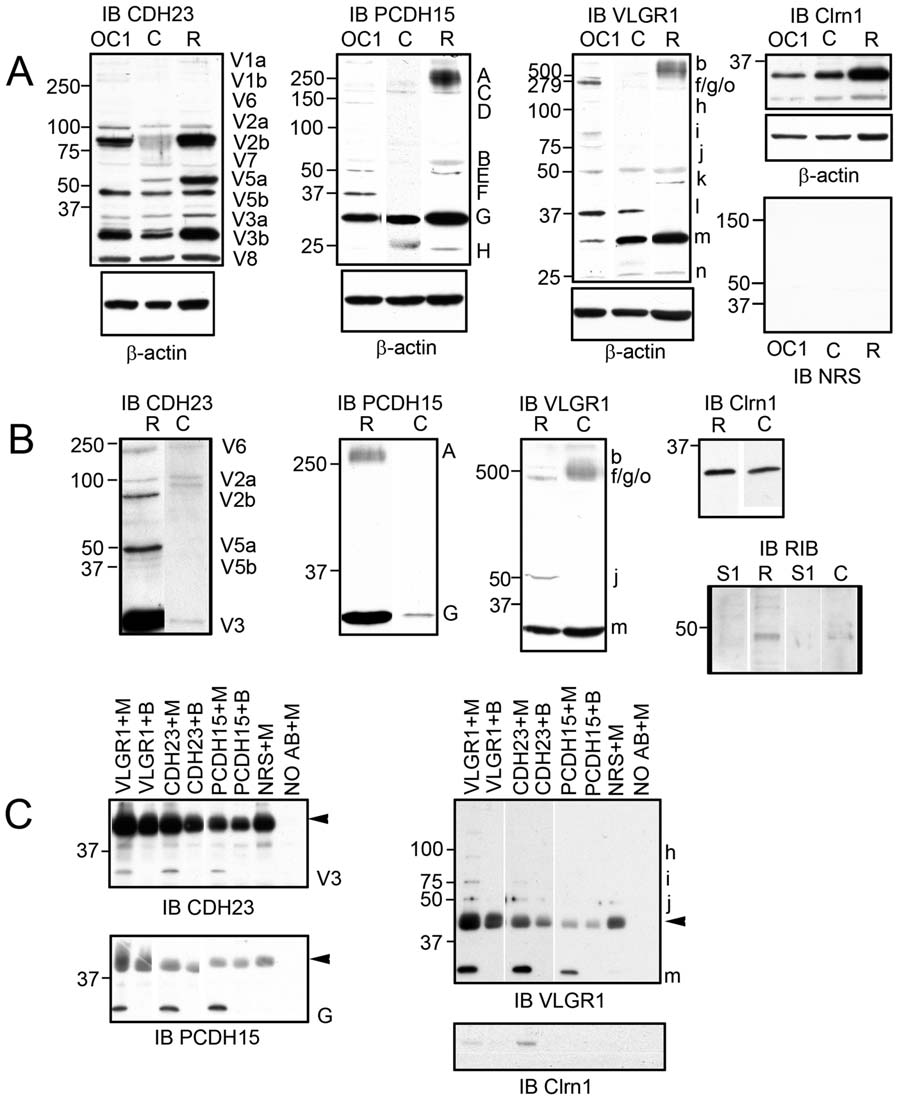
Biochemical analysis of the Usher synaptic isoforms. **Panel A:** Expression profile of the Usher proteins in differentiated UB/OC-1 cells (OC1), P3 organ of Corti (C) and neuroretina (R). β-actin was used as a loading control. NRS: normal rabbit serum (control). **Panel B:** Expression of the Usher isoforms in crude synaptosomal preparations from P3 organs of Corti (C) and neuroretina (R). The co-fractionation with RIBEYE (RIB) demonstrates the presence of specific Usher isoforms at the synapses. **Panel C:** Co-immunoprecipitation analysis in differentiated UB/OC-1 cell membrane fractions. Lanes Usher antibody+M represent specific antibody-coated beads incubated with the membrane fraction. Lanes Usher antibody+B represent specific antibody-coated beads incubated with resuspension buffer only (control 1). Lane NRS+M represents unrelated antibody-coated beads incubated with the membrane fraction (control 2). Lane NO AB+M represents un-coated beads incubated with the membrane fraction (control 3). Arrowheads denote the IgGs from the immunoprecipitation assay. Molecular weight markers are denoted to the left. Isoforms were named according to previously establish nomenclature systems Blots were cropped to show only the results relevant to this study.

To identify the specific Usher protein isoforms expressed at the basal aspect of the neurosensory epithelia, we isolated synaptosomes from P3 organs of Corti and neuroretinas, and analyzed these preparations for the presence of the Usher proteins. The expression profile of the synaptic isoforms for each Usher protein (**Fig. 9B**) was compared with whole lysates derived from the same tissues and also from differentiated UB/OC-1 cells (**Fig. 9A**).

To standardize isoform nomenclature we used the same system previously described for each Usher protein [29], [35], [47], [48], [57], [58] (**Fig. S1**). Western blot analysis of CDH23 (**Fig. 9A** **)** shows an identical pattern of isoforms in both the organ of Corti and retina and very similar to that reported by others [23], [29], [49]. We detect the full length CDH23 (isoforms “V1a” and “V1b”, 370/365 kDa), isoforms “V2a” and “V2b” and the small “synaptic” isoforms described by Lagziel et al. [29] in hair cells and photoreceptors (“V3a” and “V3b”). We also observe a weak band that we named “V6”, near the 250 kDa protein marker that may belong to the group of isoforms lacking one or two cadherin repeats [57], and a doublet at 50 kDa previously described [23], [49] and designated by us as “V5a” and “V5b” isoforms. UB/OC-1 cells only express isoform “V5b”. Two novel isoforms were detected by our antibody preparation in both sensory organs as well as in UB/OC-1 cells, “V7” near the 75 kDa protein marker and a very small one at 25 kDa designated as “V8”.

There are three common PCDH15 isoforms present in the protein lysates. The full length PCDH15 (roughly 250 kDa, isoform “A”) [35], [52], [53], an additional high molecular weight band “C” and a small isoform “G”. In retina and UB/OC-1 cells, we detect two more isoforms (“B”, “E”) around the 50 kDa marker. UB/OC-1 cells express a 150 kDa (isoform “D”), most likely one of the CD1-2 to CD1-10 isoforms (**Fig. S1**) and a 40 kDa protein isoform (“F”). These isoforms are undetectable or absent from P3 organ of Corti. PCDH15 isoform ”H” (about 25 kDa) is only present in organ of Corti and neuroretina but not in the hair cell line, suggesting that this isoform may be expressed in neurons that contact the base of hair cells and photoreceptors. Most of these PCDH15 protein isoforms have been described previously in mouse cochlea and retina at the transcript or protein level (**Fig. S1**), however the small isoforms, “E” and “F”, are novel isoforms [12], [22], [23], [35], [46], [51], [53], [59].

The VLGR1 isoform profile shows differences between P3 organs of Corti and retinas. Both tissues and the cell line express the full length VLGR1 (690 kDa, “*b”* isoform), a 50 kDa isoform “*Vlgr1j*” (**Fig. S1**), isoform “*m”* at roughly 30 kDa and a small isoform at 25 kDa (“*n”*). Organ of Corti and hair cells express a tissue specific isoform of 40 kDa (“*l”*) while neuroretina expresses an isoform slightly smaller than 50 kDa (“*k”*). The additional isoforms are only observed in the hair cell line (“*Vlgr1f/g/o”* between the 500 kDa and 279 kDa protein markers, “*Vlgr1h”* larger than the 100 kDa protein maker and *“Vlgr1i”*, a doublet at about 75 kDa), and may represent hair cell specific isoforms or low abundance isoforms undetectable in whole organ of Corti. To our knowledge this is the first evidence demonstrating the existence of small protein isoforms for VLGR1. Although several reports have been published regarding localization and alternative transcript expression for VLGR1 [15], [17], [27], [30], [48], [49], [52], [60], only one shows the presence of the full length protein in chick retina [27], there is no additional information regarding the smaller protein isoforms. By performing RACE with primers specific to exon 48, we found two novel 5′-end products (Vlgr1 f.1 of 4.9 kb and Vlgr1 g.1 of 780 bp) containing in frame favorable Kozak consensus sequences [61] and one novel 3′-end product (Vlgr1 f.2 of 800 bp) of the *Vlgr1* gene. When combined, along with the full length 5′ or 3′ terminus of *Vlgr1b*, these products can give rise to at least five different transcripts. Because the 5′ RACE products are partial sequences lacking the 3′-end, it is possible that these transcripts end in exon 52 (the novel 3′-end we characterized) or continue to exon 90, the last exon of the gene. These will give rise to 4 different transcripts (**Fig. S1**), two starting at exon 24 and ending at exon 52 or 90 (*“Vlgr1h”* and *“Vlgr1f”*, respectively*)*, and two additional transcripts starting at exon 43 and ending in one of the downstream exons (“*Vlgr1j”* and *“Vlgr1g”*). The 3′ RACE product that includes the last 3 nucleotides of intron 49 and ends in exon 52 can produce a transcript starting in exon 1 (*“Vgr1o”*) or in one of the starting sites corresponding to the Mass transcripts for which no direct association with a particular termination site has been firmly established [58]. **Figure S1** shows the putative four *Vlgr1* transcripts derived from the 5′ RACE and only one transcript derived from the combination of the 3′RACE sequence and the translational initiation site at exon 1. These five transcripts contain the complete coding sequence for EAR/EPTP domain (**Fig. S1**).

It should be noted that the isoforms discussed for CDH23, PCDH15 and VLGR1 do not represent non-specific binding of antibodies since all of them are significantly reduced in siRNA knockdown UB/OC-1 cells and by pre-adsorption of the antibodies with the peptide immunogen (**Fig. 2A** and **2B**). Because the transcripts for the small isoforms of PCDH15 (“E” and “F”) and VLGR1 (“*i*”, “k” to “n”) have not yet been described it is possible that they are originated from proteolysis of the large isoforms. However, the fact that most of them are expressed by organ of Corti, neuroretina and UB/OC-1 cells, and that some of them are present in the synaptosome preparations from the same tissues (**Fig. 9B**) supports the likelihood that these isoforms are products from yet un-described alternative transcripts or from selective proteolytic cleavage. As was shown before [19]; [25], the predominant clarin-1 isoform in all three lysates is the 30 kDa (isoform 2).

In an attempt to identify which of these isoforms are present at the neurosensory cell synapses, we employed a method that allows the isolation of the synaptosomal compartment [45]. Results from these synaptosomal preparations (**Fig. 9B**) were compared with the profile in **Fig.** **9A**. Clarin-1 and specific isoforms of CDH23, PCDH15 and VLGR1 not only are present in the sub-cellular fraction corresponding to the synaptosomes but also co-fractionate with RIBEYE (46 kDa), a specific and major component of the ribbon synapses [62]. The organ of Corti synaptosomal preparation was also positive for SNAP25, a different synaptic marker expressed in the neurosensory epithelia (**Fig. S5**) [29]. PMCA2, an apical marker for hair cells [63] was absent from this fraction but present in the supernatant S1 (**Fig.** **S5**), validating the specificity of the synaptosomal preparations. Several CDH23 isoforms are present in synaptosomal preparations from both retina and organ of Corti, including isoforms “V3”, which were previously described as synaptic [29]. Because these two isoforms do not contain the extracellular and transmembrane domains, they are cytosolic and may function as scaffold proteins through their PDZ (Postsynaptic density 95/Discs large/Zona occludens-1) domains [29]. We did not detect expression of the full length CDH23. We only observed expression of the full length PCDH15 (isoform “A*”*) in the retinal synaptosomes, possibly due to the limited starting material used for the cochlea preparation. PCDH15 isoform “G*”* is detected in synaptosomes from both the organ of Corti and the retina. The full length VLGR1 protein isoform (“*Vlgr1b”*) is present in the synaptosomes of both types of neurosensory epithelia as well as isoforms “*Vlgr1f, g or o”* and “*Vlgr1m”*. A 50 kDa isoform of VLGR1 (“*Vlgr1j”*) is also detected in the retinal preparation, but not in the organ of Corti preparation. Clarin-1 (isoform 2) is observed in the synaptosomes from both types of neurosensory cells. The differences in the expression profile between retinal and cochlear synaptosomes may reflect tissue specificity or/and differences in protein abundance between tissues.

Due to the limited amount of material that we can obtain from mouse cochlea or retina, we utilized a membrane-enriched fraction from differentiated UB/OC1 cells as a platform for biochemical analysis of native Usher protein interactions. We analyzed the interactions between CDH23, PCDH15, VLGR1 and clarin-1 using a co-immunoprecipitation approach (**Fig. 9C**). We observed interactions between the synaptic isoform of CDH23 (“V3*”*) and specific isoforms recognized by anti-PCDH15 and anti-VLGR1. The small isoform of PCDH15 (“G*”*) interacts with CDH23 and VLGR1 while the small isoform of VLGR1 (“*Vlgr1m”*) associates with CDH23 and PCDH15. There is also a specific interaction between VLGR1 isoforms “*Vlgr1i”* and “*Vlgr1j*” and CDH23, and within VLGR1 protein isoforms (between “*Vlgr1h”, “Vlgr1i”*, “*Vlgr1j”* and “*Vlgr1m”*). Isoform 2 of clarin-1 shows association with CDH23 and VLGR1 in UB/OC-1 membrane fractions. All these interactions are specific as shown by the controls for specificity run in parallel for each experiment.

### Usher mutant mice have altered cochlear synapses

**Figure 10:**
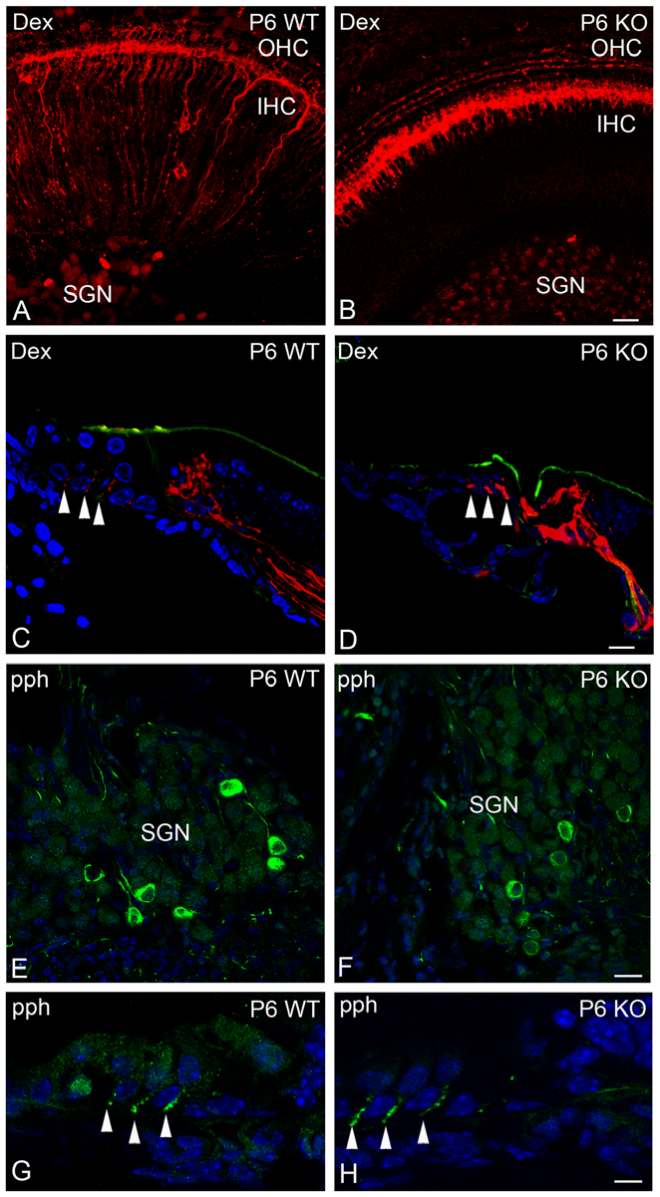
Clrn1−/− mouse has immature type I afferent fibers. P6 WT (**A,** **C, E, G**) or *Clrn1−/−* (**B, D, F, H**) whole mount organ of Corti (**A–B**) or cochlea cross-sections (**C–H**) were labeled with the neuronal tracer (**A–D**, red) and counter-stained with phalloidin for F-actin (green, **C–D**) or with peripherin (pph, green, **E–H**). Arrowheads: specific staining at the base of the OHCs. Scale bars: **A–B**: 20 µm, **C–D**: 10 µm, **E–F**: 15 µm and **G–H**: 6 µm.

To test the potential function of the synaptic Usher protein complex in synaptic maturation *in vivo*, we analyzed the morphology of the developing neuronal fibers and synaptic contacts in WT and *Clrn1−/−* mice [31], [33]. Staining of type I afferent fibers with a fluorescent neuronal tracer suggests a defect in neuronal fiber maturation at P6 in the *Clrn1*−/− cochlea compare with the WT (**Fig. 10A–D**). While the type I innervation at the base of OHCs is barely detectable in P6 WT (**Fig. 10A** and **10C**), it is still readily visible in the mutant mouse (**Fig. 10B** and **10D**) and similar to the innervation pattern found in WT mice at early ages (data not shown) [40]. No alterations were found in the type II SGNs and neuronal fibers at the confocal level between WT and mutant cochleae (**Fig.10E–H**).

**Figure 11:**
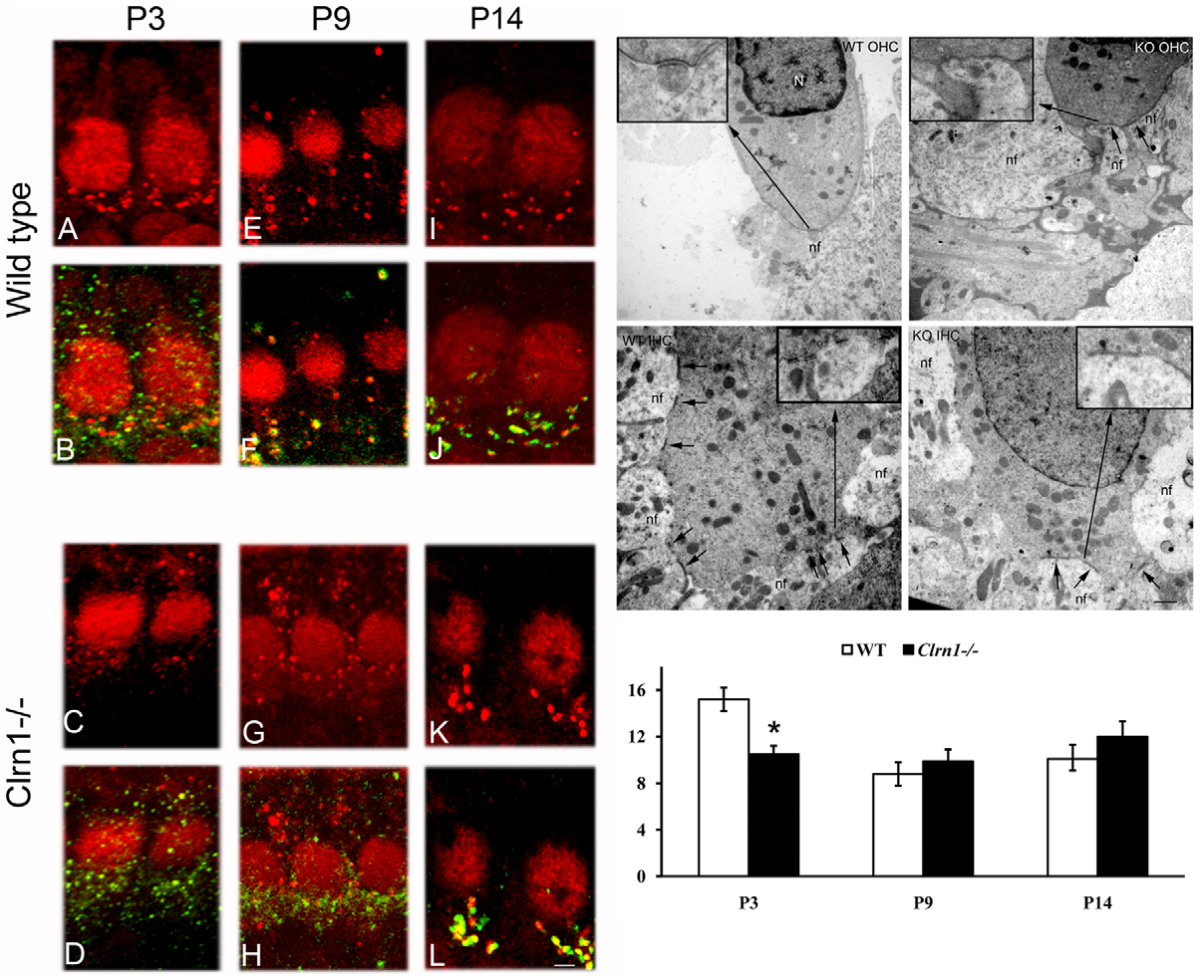
Clrn1−/− mouse has immature synaptic contacts. P3 (**A–D**), P9 (**E–H**) and P14 (**I–L**) IHC ribbon synapses immunostained for the pre-synaptic marker RIBEYE (red) and the post-synaptic marker GluR2/3 (green). Bar graph: Quantitative analysis of the ribbons in WT and *Clrn1−/−* IHCs. Student's t test shows significant differences at P3 only (asterisk). **M–P:** Ultrastructural analysis of WT (**M, O**) and *Clrn1−/−* (**N, P**) P9 synapses at the base of OHCs (**M–N**) and IHCs (**O–P**). Small arrows denote synaptic contacts between hair cells and neuronal fibers. Insets: magnification of pointed area. N: nucleus. nf: neuronal fiber.. Scale bars. **A–L:** 2 µm, **N–Q**: 500 nm.

The IHC ribbon synapses in the *Clrn1*−/− cochlea were analyzed by confocal microscopy using pre-synaptic (RIBEYE) and post-synaptic (GluR2/3) markers (**Fig. 11A–L**) [37]. At P3 the synapses are very immature with the classic juxtaposed pair (RIBEYE/GluR2/3) absent from the base of WT and mutant IHCs (**Fig. 11A–D**). However, the number of ribbons is significant reduce in the *Clrn1−/−* cochlea compare with the WT. At P9 the WT ribbon synapses can be easily identified as a juxtaposed pair of immunofluorescence spots (**Fig. 11E–F**), in the mutant the RIBEYE/GluR2/3 pair is still absent (**Fig. 11G–H**). The number of ribbons is similar between mutant and WT (bar graph) but only due to the decrease that normally occurs during cochlea maturation. The GluR2/3 immunoreactivity in the *Clrn1−/−* (green) shows a confluent pattern enclosing the entire basolateral surface of the IHCs characteristic of immature ribbon synapses (**Fig. 11B**) [37], [64]. By P14 the juxtaposed pre- and post-synaptic pairs have already been formed at the base of the mutant IHCs (**Fig. 11K–L**), demonstrating a delay in synaptic maturation in the *Clrn1−/−* cochlea. No significant differences were observed in the number of ribbons per IHC between WT and *Clrn1−/−* at P9 and P14 (bar graph, P3: 15.2±1.0 *versus* 10.5±0.7, n = 4 cochleae; P9: 8.8±1.0 *versus* 9.9±1.2, n = 3 WT and 4 KO cochleae; P14: 10.1±0.9 *versus* 12.0±1.3, n = 5 WT and 4 KO cochleae). Ultrastructural analysis of the synapses also shows abnormalities in P9 *Clrn1*−/− mice (**Fig. 11M–P** and *insets*). We observed multiple synaptic contacts in the *Clrn1*−/− OHCs compared with the WT (**Fig. 11M–N**). These synapses are less defined and underdeveloped, more reminiscent of the WT at an earlier time point. At the base of the IHCs (**Fig. 11O–P**), the synaptic densities also display qualitative differences. In the WT mice the synapses present a confined electron dense area and show both pre- and post-synaptic densities (**Fig. 11O** *inset*). In the mutant mice (**Fig. 11P** *inset*), the synaptic densities are diffuse and more broadly dispersed, with neuronal contacts spanning longer consecutive lengths along the hair cell plasmalemma, which is a typical configuration of an immature organ of Corti [6]. This result, as well as the increased number and area of the synaptic contacts observed ultrastructurally at P9 in the *Clrn1−/−* are characteristic of immature WT hair cells [60], and suggest a defect in synaptic maturation.

The PCDH15 mutant mouse *Ames waltzer^av3J^* was also used in these studies (**Fig. S6**). Mutant animals still express PCDH15 transcripts and proteins (data not shown). The analysis of the ribbon synapses in P9 *Ames waltzer^av3J^* animals shows differences in synaptic protein expression between mutant (**Fig. S6**) and WT animals (**Fig. 11E–F**). Consistent with the *Clrn1−/−* mouse, we observed an altered distribution of the post-synaptic marker GluR2/3 by confocal immunostaining, suggesting that the delay in synaptic maturation may be a more general feature shared by the different Usher models, and further supporting a functional role for the novel complex of small Usher protein isoforms in hair cell synaptic maturation.

## Discussion

Since the sequential discovery of the genes associated with Usher syndrome, there have been many reports describing expression of the Usher related proteins in the affected organs, the eye and inner ear [20], [21], [65]. Regarding the latter, most of the immunolocalization and morphological studies have been focused at the apical aspect of vestibular and cochlear hair cells, probably because of the structural abnormalities observed in the stereocilia bundles for the different mouse models [25]–[27]; [31], [32]. The fact that vision is also affected and that the retina (the main affected tissue in the eye) is devoid of stereocilia-like (actin paracrystal) structures led us to examine Usher protein function at the ribbon synapses, highly specialized structures present in both photoreceptors and hair cells, where many of the Usher proteins have been localized [19], [22], [29], [30], [45]. A developmental survey for Usher protein expression focused our initial studies on CDH23, PCDH15 (two cadherins involved in cell-cell adhesion contacts) and VLGR1 (a G-protein coupled receptor involved in neurogenesis) [17], [18], [60], [66], [67]. We developed and characterized antibodies for these three Usher proteins [24], [33], [34]. Due to the presence of multiple isoforms generated by alternative splicing and/or by the use of alternative promoters and splice sites [14], [24], [28], [29], [35], [46]–[49], [51], [59], [68], most of the Usher mouse models available are not true knockouts but spontaneous or induced mutants that either lack some of these isoforms or express dysfunctional proteins [29], [46]–[49]. Just recently, the expression of several PCDH15 isoforms in the stereocilia were described using the *Ames waltzer* mouse model *av6J*, that carries a presumptive in-frame deletion in exon 22 [69]. The use of antibodies directed against the three different cytoplasmic domains of PCDH15 (CD1, CD2 and CD3) demonstrated normal immunostaining at the stereocilia. However the use of an extracellular domain-directed antibody showed a marked decreased in PCDH15 expression, demonstrating that the selection of the domain used to raise antibodies is a critical step for isoform detection [69]. This concept is further validated by our demonstration of apical stereociliary immunostaining for VLGR1 hair cells isolated from P30 mice (**Fig. S2**), which suggests that a previously undescribed isoform of VLGR1 containing the EAR domain, but not the carboxyl-terminal domain is present in mature hair cell stereocilia.

Therefore, to establish the isoform specificity of our antibody preparations, we used two different approaches: 1) western blot analysis of P3 inner ear and neuroretina with the affinity purified primary antibody (A–P) or the flow through (F–Th) following pre-adsorbtion of antibodies with immobilized peptide immunogen and 2) transient knock down studies of the Usher transcripts in differentiated UB/OC-1 cells using siRNAs derived from the DNA sequences comprising the peptide immunogens (**Figs. 1** **,** **2** and **Table S1**). Bands observed on western blots using affinity purified antibodies were markedly reduced or absent when duplicate blots were probed with the flow through following affinity purification on immobilized peptide immunogens. UB/OC-1 cells showed differences in the efficiency of knockdown for all the Usher transcript isoforms within a given Usher protein with a reduction in expression between 20% to 100%, likely due to differences in the efficacy of the siRNAs employed.

We were able to establish a spatiotemporal pattern of expression for the Usher proteins at the apical and basal aspects of the hair cells and in the neuronal terminals, between P1 and P14 (**Fig. 3**). Basally, the pattern is similar to the one described for clarin-1 [16], [19] and parallels the developmental window when synaptic remodeling takes place at the base of the OHCs during the first post-natal weeks in mouse cochlea [5], [6], [8], [40].

We show, for the first time, that CDH23, PCDH15, VLGR1 and clarin-1 are also expressed by both types of afferent fibers (**Fig. 6** **–** **7**), with very prominent expression in a sub-population of type I afferent neurons. The expression is not restricted to the neuronal terminals but is observed throughout the neuronal fibers to the SGN cell bodies. We also observed weak and diffuse immunostaining in the cell bodies and at brighter spots of immunostaining at the base of isolated hair cells from P3 mice **(** **Fig. 5** **)**. Although this basal immunoreactivity was observed repetitively in the different hair cell preparations, we cannot rule out the possibility that this staining may be due to post-synaptic fibers that remain attached after the isolation procedure. We feel this is unlikely, however, given the clear co-localization of these proteins with myosin7A in the isolated hair cell preparations **(** **Fig. 5** **)**. Cochlea cross-sections **(** **Fig. 4** **)** also show diffuse Usher protein expression in the hair cell bodies that is similar to that observed for other synaptic proteins at early developmental stages. This may reflect the active protein trafficking that is taking place during this maturational process [70]–[75].

In mature mouse cochlea (**Figs. 8** **,** **S2** and **S4**) the Usher proteins are still present post-synaptically at the base of IHCs and in the contacting type I afferent fibers. This suggests two different roles for the Usher proteins at the synapses, i) an early role that may involve type I afferent synaptic maturation and ii) a later role most likely related to maintenance of the afferent fibers and their synaptic contacts in the adult cochlea (the isoforms involved in these two functions may or may not be the same ones). While mature hair cells show absence of basal immunostaining for the Usher proteins (with the exception of PCDH15), their expression persists at the stereocilia level (**Fig. S2**). In the case of VLGR1, a component of the transient ankle links involved in hair bundle development [27], [52], we were able to detect strong expression in the apical region of the stereocilia at P30 suggesting an additional and novel role for EAR/EPTP domain-containing VLGR1 isoform(s) in mature stereocilia.

There are several lines of evidence reinforcing our notion of a role for these Usher proteins in synaptogenesis. First, the VLGR1 synaptic isoforms recognized by anti-VLGR1 contain the EAR/EPTP domain. This domain defines a family of proteins implicated in epileptic disorders and, in the case of VLGR1, play a key role in a Mendelian type of audiogenic epilepsy [17], [18], [67]. Second, clarin-1 is a tetraspanin with a high degree of sequence similarity to stargazin, another tetraspanin of the same superfamily. Because stargazin is involved in synaptic plasticity and maintenance, it has been suggested a similar synaptic function for clarin-1 [16], [76], [77]. Third, the *Clrn1−/−* mouse shows a deficit in afferent neuronal activation [25]. The presence of the same small Usher isoforms at the synapses of both type of neurosensory epithelia and the interactions observed between each other in UB/OC-1 cells (**Fig.** **9**) strongly supports the existence of a functional Usher synaptic complex *in vivo*, with a potential role in synaptogenesis. Although we cannot completely rule out an indirect effect of the Usher proteins in synaptic maturation, the evidence presented here, along with the recent novel findings of a role for harmonin (isoform a) in the regulation of Ca_V_1.3 channel expression and function at the hair cell surface [78], argue in favor of a direct role for some Usher protein isoforms in synaptic maturation.

In the case of VLGR1 we characterized novel transcripts containing the complete coding sequence for the EAR/EPTP domain (**Fig. S1**) and may account for some of the protein isoforms detected in **Fig. 9**. However, we did not observe any small transcripts corresponding to the small bands detected by western blot (isoforms “*i*”, “*k*” to “*n*”). These likely represent still un-characterized transcripts or, as we suggested above, selective proteolytic cleavage products.

We observed defects in synaptic maturation in *Clrn1−/−* and altered synapses in the *Ames waltzer^av3J^* mice (**Figs. 10** **–** **11** **, S6**). The abnormalities we documented at the mutant ribbon synapses are strikingly similar to those observed in athyroid mouse models where synaptic maturation is impaired [37]. Although the Usher proteins are also present in type II afferent fibers it seems that the main defect in the *Clrn1−/−* mouse emanates from the type I afferent neurons as these are the ones showing a maturational delay (**Fig. 10**). Although we cannot completely exclude the involvement of clarin-1 and PCDH15, as isolated proteins, in hair cell synaptic development, we believe this is unlikely due the tremendous amount of work showing the existence of an “Usher interactome” [20], [21], [24]; [26], [30], [34], [65]; [79]–[82].

In conclusion, we show that specific Usher isoforms are expressed pre- and post-synaptically in cochlear hair cells. There is prominent expression of the Usher proteins in a sub-population of type I afferent neurons that at P3 innervate both types of hair cells but at P30 innervate only IHCs. At the synapses, our results suggest the existence of a novel Usher complex comprised mostly of small isoforms. The absence of one of the components of the complex, i.e. clarin-1 or PCDH15, results in defective synaptic maturation, providing new evidence for a role of Usher proteins at the synapses. This function may be direct through the interaction and/or modulation of specific synaptic proteins as was recently described for harmonin [78] or indirect through a more general mechanism that ultimately affects the maturation and function of the hair cell synapses. Altogether, the data suggest that the synaptic Usher protein complex may provide signals that direct the synaptic remodeling that occurs during development in cochlear hair cells. Investigation of whether similar defects in synaptic development/structure/function are also observed in the retina of Usher mouse models is needed. This work underscores the fact that there are a large number of distinct Usher protein isoforms that form specific complexes, likely many, as is the case for the synaptic complex, with unexpected compositions and functions. Thus it is likely that current views regarding the function of Usher proteins in hair cells and photoreceptors are oversimplified. We propose that the Usher protein complexes function as mediators of protein trafficking, docking, and/or membrane fusion, likely influencing both protein and membrane dynamics at both the apical and basal aspects of neurosensory epithelial cells.

## Supporting Information

Figure S1 **Schematic representation of the Usher isoforms relevant to this work**. **VLGR1 isoforms:** Predicted structure and domains of the full length VLGR1 (Vlgr1b). CalX-β: Ca^2^+-binding domain of the Na^+^/Ca^2+^ exchanger molecule. LamG/TspN/PTX: laminin-G/thrombospondin-N/pentraxin homology domains. EAR/EPTP: epilepsy-associated repeat/epitempin repeat GPS: G-protein-couple receptor proteolytic site TM: transmembrane domain. Cyt: cytoplasmic domain. The new transcripts obtained by RACE are also included (Vlgr1f, Vlgr1g, Vlgr1h, Vlgr1j and Vlgr1o). 5′RACE products: 4.9-kb and 780-bp products comprising exons 24 to 48 and 43 to 48, respectively. These partial 5′ transcripts were combined with the rest of the sequence for Vlgr1b or with the novel 3′RACE product, generating Vlgr1f, Vlgr1h, Vlgr1g and Vlgr1j complete transcripts. 3′RACE product: 800-bp product comprising exons 48 to 52. This partial 3′ transcript was combine with the novel 5′RACE products and with the beginning of the transcript for Vlgr1b, generating Vlgr1h, Vlgr1j and Vlgr1o complete transcripts. The protein domains for each new isoform and their estimated molecular weights are also included. **PCDH15 isoforms:** The full length PCDH15-CD1 isoform (CD1-1 or isoform A) contains 11 cadherin repeats, a transmembrane domain (TM) and the cytoplasmic domain 1 (CD1) that includes the PDZ binding sequence “STSL”. Several isoforms lacking a small part of the full length PCDH15-CD1 have also been described (isoforms CD1-2 to CD1-10). Isoform B containing only one cadherin repeat and several identified but still uncharacterized isoforms containing part of the extracellular domain (D, G and H). Apparent or estimated molecular weights are included. **CDH23 isoforms:** The full length CDH23 (V1) contains 27 cadherin repeats, a transmembrane domain (TM) and a cytoplasmic domain (Cyt) with or without the coding sequence present in exon 68 (red box). The CDH23 V2 isoforms only contain 7 cadherin repeats and the CDH23 V3 isoforms are cytosolic as they only include the cytoplasmic domain and a 7 unique amino acid sequence (black box). Additional CDH23 isoforms have been identified at the transcript (V4 and V6) or protein (V5) level. The apparent or estimated molecular weights for all these isoforms have been included. *Transcripts and putative protein isoforms characterized in this work. §Isoforms characterized by others but named by us, following the already established nomenclature system.
(TIF)

Figure S2 Isolated hair cells from P30 organs of Corti were immunostained for the CDH23 (**A, a′, a″**), VLGR1 (**B, b′, b″**), PCDH15 (**C, c′, c″**) and clarin-1 (**D, d′, d″**) (green), the hair cell marker myosin7A (magenta) and counter-stained with phalloidin (red). Asterisks: apical staining and co-localization with phalloidin. Scale bar: **A–D:** 2.5 µm; **a′–d″:** 5 µm.
(TIF)

Figure S3 **Pre- and post-synaptic expression of CDH23, PCDH15, VLGR1 and clarin-1 in P3 cochleae.** Single plane images from P3 cochlea cross-sections immunostained for the Usher proteins (magenta) and the pre-synaptic marker RIBEYE (green). CDH23 (**A–B**); PCDH15 (**C–D**); VLGR1 (**E–F**) and clarin-1 (**G–H**). Arrowheads denote basal pre-synaptic co-localization in OHCs. Scale bar: 5 µm.
(TIF)

Figure S4 **Expression of the Usher proteins in P60 type I afferent neurons.** Cochlea cross-sections immunostained for CDH23 (**A–B**), PCDH15 (**C–D**), VLGR1 (**E–F**) and clarin-1 (**G–H**), showing expression of the Usher proteins in the type I afferent terminals that synapse the IHCs (**A, C, D, E, G**) and corresponding SGNs (**B, D, F, H**) . Scale bar: **A, C, E, G:** 10 µm. **B, D, F, H:** 25 µm.
(TIF)

Figure S5 **Western blot analysis of synaptosomal preparation from P3 organ of Corti.** **Left**: the synaptic marker SNAP25 was used to demonstrate the presence of the synaptosomal preparation in P2 (see Materials and Methods). Note that the apparent molecular mass of SNAP25 is bigger than expected (25 kDa) as native conditions were used. **Right**: the apical marker, PMCA2, was used to demonstrate distinct sub-cellular fractionation. PMCA2 is present in S1 and absence from P2 where the crude synaptosomes fractionate.
(TIF)

Figure S6 **Ames waltzer^av3J^ mouse has immature synaptic contacts.** P9 PCDH15 mutant IHC ribbon synapses immunostained for the pre-synaptic marker RIBEYE (red) and the post-synaptic marker GluR2/3 (green). Scale bar: 3 µm.
(TIF)

Table S1 Description of the mouse fusion peptides used to generate the corresponding Usher antibodies. siRNA and qRT-PCR primers used for specificity control experiments are also included. Immunogen region for each Usher protein are show below the table.
(TIF)

## References

[1] Fuchs PA, Glowatzki E, Moser T (2003). The afferent synapse of cochlear hair cells.. Curr Opin Neurobiol.

[2] Moser T, Neef A, Khimich D (2006). Mechanisms underlying the temporal precision of sound coding at the inner hair cell ribbon synapse.. J Physiol.

[3] Dannhof B, Bruns V (1993). The innervation of the organ of Corti in the rat.. Hear Res.

[4] Raphael Y, Altschuler RA (2003). Structure and innervation of the cochlea.. Brain Res Bull.

[5] Ruel J, Wang J, Rebillard G, Eybalin M, Lloyd R (2007). Physiology, pharmacology and plasticity at the inner hair cell synaptic complex. Hea.. Res.

[6] Sobkowicz H, Rose J, Scott G, Slapnick S (1982). Ribbon synapses in the developing intact and culture organ of Corti in the mouse.. J Neurosci.

[7] Sobkowicz HM, Rose JE, Scott GL, Levenick CV (1986). Distribution of synaptic ribbons in the developing organ of Corti.. J Neurocytol.

[8] Pujol R (1985). Morphology, synaptology and electrophysiology of the developing cochleae.. Acta Otolaryngo.

[9] Simmons D (1994). A transient afferent innervation of outer hair cells in the postnatal cochlea.. NeuroReport.

[10] Boughman JA, Vernon M, Shaver KA (1983). Usher syndrome: definition and estimate of prevalence from two high risk populations.. J Chronic Dis.

[11] Hallgren B (1959). Retinitis pigmentosa combined with congenital deafness; with vestibule-cerebellar ataxia and neural abnormality in a proportion of cases.. Acta Psychiatr Scand.

[12] Alagramam KN, Yuan H, Kuehn MH, Murcia CL, Wayne S (2001). Mutations in the novel protocadherin PCDH15 cause Usher syndrome type 1F.. Hum Mol Genet.

[13] Bolz H, von Brederlow B, Ramírez A, Bryda EC, Kutsche K (2001). Mutation of CDH23, encoding a new member of the cadherin gene family, causes Usher syndrome type 1D.. Nat Genet.

[14] Di Palma F, Pellegrino R, Noben-Trauth K (2001). Genomic structure, alternative splice forms and normal and mutant alleles of cadherin 23 (Cdh23).. Gene.

[15] Weston MD, Luijendijk MWJ, Humphrey KD, Moller C, Kimberling WJ (2004). Mutations in the VLGR1 gene implicate G-protein signaling in the pathogenesis of Usher syndrome type II.. Am J Hum Genet.

[16] Adato A, Vreugde S, Joensuu T, Avidan N, Hamalainen R (2002). USH3A transcripts encode clarin-1, a four-transmembrane-domain protein with a possible role in sensory synapses.. Eur J Hum Genet.

[17] Skradski SL, Clark AM, Jiang H, White HS, Fu Y-H (2001). A novel gene causing a mendelian audiogenic mouse epilepsy.. Neuron.

[18] Staub E, Perez-Tur J, Siebert R, Nobile C, Moschonas NK (2002). The novel EPTP repeat defines a superfamily of proteins implicated in epileptic disorders.. Trends Biochem Sci.

[19] Zallocchi M, Meehan DT, Delimont D, Askew C, Garige S (2009). Localization and expression of clarin-1, the Clrn1 gene product, in auditory hair cells and photoreceptors.. Hear Res.

[20] Kremer H, van Wijk E, Märker T, Wolfrum U, Roepman R (2006). Usher syndrome: molecular links of pathogenesis, proteins and pathways.. Hum Mol Genet.

[21] Petit C (2001). Usher syndrome: from genetics to pathogenesis. Annu. Rev.. Genomics Hum Genet.

[22] Reiners J, Maerker T, Jurgens K, Reidel B, Wolfrum U (2005). Photoreceptor expression of the Usher syndrome type 1 protein protocadherin 15 (USH1F) and its interaction with the scaffold protein harmonin (USH1C).. Mol Vis.

[23] Kazmierczak P, Sakaguchi H, Tokita J, Wilson-Kubalek EM, Milligan RA (2007). Cadherin 23 and protocadherin 15 interact to form tip-link filaments in sensory hair cells.. Nature.

[24] Zallocchi M, Sisson JH, Cosgrove D (2010). Biochemical characterization of native Usher protein complexes from a vesicular subfraction of tracheal epithelial cells.. Biochem.

[25] Geng R, Geller SF, Hayashi T, Ray CA, Reh TA (2009). Usher syndrome IIIA gene clarin-1 is essential for hair cell function and associated neural activation.. Hum Mol Genet.

[26] Lefèvre G, Michel V, Weil D, Lepelletier L, Bizard E (2008). A core cochlear phenotype in USH1 mouse mutants implicates fibrous links of the hair bundle in its cohesion, orientation and differential growth.. Development.

[27] McGee J, Goodyear RJ, McMillan DR, Stauffer EA, Holt JR (2006). The very large G-protein-coupled receptor VLGR1: a component of the ankle link complex required for the normal development of auditory hair bundles.. J Neurosci.

[28] Siemens J, Lillo C, Dumont RA, Reynolds A, Williams DS (2004). Cadherin 23 is a component of the tip link in hair-cell stereocilia.. Nature.

[29] Lagziel A, Overlack N, Bernstein SL, Morell RJ, Wolfrum U (2009). Expression of cadherin 23 isoforms is not conserved: implications for a mouse model of Usher syndrome type 1D.. Mol Vis.

[30] van Wijk E, van derZwaag B, Peters T, Zimmermann U, TeBrinke H (2006). The DFNB31 gene product whirlin connects to the Usher protein network in the cochlea and retina by direct association with USH2A and VLGR1.. Hum Mol Genet.

[31] Geller SF, Guerin KI, Visel M, Pham A, Lee ES (2009). CLRN1 is nonessential in the mouse retina but is required for cochlear hair cell development.. PLoS Genet.

[32] Senften M, Schwander M, Kazmierczak P, Lillo C, Shin JB (2006). Physical and functional interaction between protocadherin 15 and myosin VIIa in mechanosensory hair cells.. J Neurosci.

[33] Cosgrove D, Zallocchi M (2010). Clarin-1 protein expression in photoreceptors.. Hear Res.

[34] Maerker T, van Wijk E, Overlack N, Kersten FF, McGee J (2008). A novel Usher protein network at the periciliary reloading point between molecular transport machineries in vertebrate photoreceptor cells.. Hum Mol Genet.

[35] Ahmed ZM, Goodyear R, Riazuddin S, Lagziel A, Legan PK (2006). The tip-link antigen, a protein associated with the transduction complex of sensory hair cells, is protocadherin-15.. J Neurosci.

[36] Soni LE, Warren CM, Bucci C, Orten DJ, Hasson T (2005). The unconventional myosin-VIIa associates with lysosomes.. Cell Motil Cytoskeleton.

[37] Sendin G, Bulankina AV, Riedel D, Moser T (2007). Maturation of ribbon synapses in hair cells is driven by thyroid hormone.. J Neurosci.

[38] Legendre K, Safieddine S, Kussel-Andermann P, Petit C, El-Amraoui A (2008). αII-βV spectrin bridges the plasma membrane and cortical lattice in the lateral wall of the auditory outer hair cells.. J Cell Sci.

[39] Boyer S, Ruel J, Puel JL, Chabbert C (2004). A procedure to label inner ear afferent nerve endings for calcium imaging.. Brain Res Brain Res Protoc.

[40] Huang LC, Thorne PR, Housley GD, Montgomery JM (2007). Spatiotemporal definition of neurite outgrowth, refinement and retraction in the developing mouse cochlea.. Development.

[41] Rivolta MN, Grix N, Lawlor P, Ashmore JF, Jagger DJ (1998). Auditory hair cell precursors immortalized from the mammalian inner ear.. Proc Biol Sci.

[42] Rivolta MN, Halsall A, Johnson CM, Tones MA, Holley MC (2002). Transcript profiling of functionally related groups of genes during conditional differentiation of a mammalian cochlear hair cell line.. Genome Res.

[43] Le-Niculescu H, Niesman I, Fischer T, DeVries L, Farquhar MG (2005). Identification and characterization of GIV, a novel Galpha i/s-interacting protein found on COPI, endoplasmic reticulum-Golgi transport vesicles.. J Biol Chem.

[44] Bradford MM (1976). A rapid and sensitive method for the quantitation of microgram quantities of protein utilizing the principle of protein-dye binding.. Anal Biochem.

[45] Reiners J, Reidel B, El-Amraoui A, Boëda B, Huber I (2003). Differential distribution of harmonin isoforms and their possible role in Usher-1 protein complexes in mammalian photoreceptor cells. Invest.. Ophthalmol Vis Sci.

[46] Alagramam K, Murcia C, Kwon H, Pawlowski K, Wright C (2001). The mouse Ames waltzer hearing-loss mutant is caused by mutation of *Pcdh15*, a novel protocadherin gene.. Nat Genet.

[47] Lagziel A, Ahmed ZM, Schultz JM, Morell RJ, Belyantseva IA (2005). Spatiotemporal pattern and isoforms of cadherin 23 in wild type and waltzer mice during inner ear hair cell development.. Dev Biol.

[48] Yagi H, Takamura Y, Yoneda T, Konno D, Akagi Y (2005). Vlgr1 knockout mice show audiogenic seizure susceptibility.. J Neurochem.

[49] Yagi H, Tokano H, Maeda M, Takabayashi T, Nagano T (2007). Vlgr1 is required for proper stereocilia maturation of cochlear hair cells.. Genes cells.

[50] Webb S, Grillet N, Andrade L, Xiong W, Swarthout L (2011). Regulation of PCDH15 function in mechanosensory hair cells by alternative splicing of the cytoplasmic domain.. Development.

[51] Alagramam KN, Miller ND, Adappa ND, Pitts DR, Heaphy JC (2007). Promoter, alternative splice forms and genomic structure of protocadherin 15.. Genomics.

[52] Michalski N, Michel V, Bahloul A, Lefèvre G, Barral J (2007). Molecular characterization of the ankle-link complex in cochlear hair cells and its role in the hair bundle functioning.. J Neurosci.

[53] Ahmed ZM, Riazuddin S, Ahmed J, Bernstein SL, Guo Y (2003). PCDH15 is expressed in the neurosensory eptithelium of the eye and ear and mutant alleles are responsible for both USH1F and DFNB23.. Hum Mol Genet.

[54] Hasson T, Gillespie P, Garcia J, MacDonald R, Zhao Y (1997). Unconventional myosins in inner-ear sensory epithelia.. J Cell Biol.

[55] Gil-Loyzaga P, Pujol R (1990). Neurotoxicity of kainic acid in the rat cochlea during early developmental stages.. Eur Arch Otorhinolaryngol.

[56] Barclay M, Julien J, Ryan A, Housley G (2010). Type III intermediate filament peripherin inhibits neuritogenesis in type II spiral ganglion neurons in vitro.. Neurosci Lett.

[57] Ahmed Z, Kjellstrom S, Haywood-Watson R, Bush R, Hampton L (2008). Double homozygous waltzer and Ames waltzer mice provide no evidence of retinal degeneration.. Mol Vis.

[58] McMillan R, White P (2004). Loss of the transmembrane and cytoplasmic domains of the very large G-protein-coupled receptor-1 (VLGR1 or Mass1) causes audiogenic seizures in mice.. Mol Cell Neurosci.

[59] Haywood-Watson RII, Ahmed Z, Kjellstrom S, Bush R, Takada Y (2006). Ames Waltzer deaf mice have reduced electroretinogram amplitudes and complex alternative splicing of Pcdh15 transcripts.. Invest Ophthalmol Vis Sci.

[60] McMillan DR, Kayes-Wandover KM, Richardson JA, White PC (2002). Very large G protein-coupled receptor-1, the largest known cell surface protein, is highly expressed in the developing central nervous system.. J Biol Chem.

[61] Kozak M (1996). Interpreting cDNA sequences: some insights from studies on translation.. Mamm Genome.

[62] Zanazzi G, Matthews G (2009). The molecular architecture of ribbon presynaptic terminals.. Mol Neurobio.

[63] Grati M, Scheneider ME, Lipkow K, Strehler EE, Wenthold RJ (2006). Rapid turnover of stereocilia membrane proteins: evidence from the trafficking and mobility of plasma membrane Ca^2^+-ATPase 2.. J Neurosci.

[64] Nemzou RM, Bulankina AV, Khimich D, Giese A, Moser T (2006). Synaptic organization in CaV1.3Ca^2+^ channel deficient cochlear hair cells.. Neurosci.

[65] Reiners J, Nagel-Wolfrum K, Jürgens K, Märker T, Wolfrum U (2006). Molecular basis of human Usher syndrome: deciphering the meshes of the Usher protein network provides insights into the pathomechanisms of the Usher disease.. Exp Eye Res.

[66] Nikkila H, McMillan R, Nunez B, Pascoe L, Curnow K (2000). Sequence similarities between a novel putative G protein-coupled receptor and Na^+^/Ca^2+^ exchangers define a cation binding domain.. Mol Endo.

[67] Scheel H, Tomiuk S, Hofmann K (2002). A common protein interaction domain links two identified epilepsy genes.. Hum Mol Genet.

[68] Ahmed ZM, Riazuddin S, Aye S, Ali RA, Venselaar H (2008). Gene structure and mutant alleles of PCDH15: nonsyndromic deafness DFNB23 and type 1 Usher syndrome.. Hum Genet.

[69] Alagramam K, Goodyear R, Geng R, Furness D, van Aken A (2011). Mutations in protocadherin 15 and cadherin 23 affect tip links and mechanotransduction in mammalian sensory hair cells.. PLoS one.

[70] Heidrych P, Zimmermann U, Breβ A, Pusch CM, Ruth P (2008). Rab8b GTPase, a protein transport regulator, is an interacting partner of otoferlin, defective in a human autosomal recessive deafness form.. Hum Mol Gen.

[71] Heidrych P, Zimmermann U, Kuhn S, Franz C, Engel J (2009). Otoferlin interacts with myosin VI: implications for maintenance of basolateral synaptic structure of the inner hair cell.. Hum Mol Gen.

[72] Beurg M, Michalski N, Safieddine S, Bouleau Y, Schneggenburger R (2010). Control of exocytosis by synaptotagmins and otoferlin in auditory hair cells.. J Neurosci.

[73] Knirsch M, Brandt N, Braig C, Kuhn S, Hirt B (2007). Persistence of Ca_V_1.3 Ca^2^+ channels in mature outer hair cells supports outer hair cell afferent signaling.. J Neurosci.

[74] Roux I, Hosie S, Johnson SL, Bahloul A, Cayet N (2009). Myosin VI is required for the proper maturation and function of inner hair cell ribbon synapses.. Hum Mol Gen.

[75] Safieddine S, Wenthold R (1999). SNARE complex at the ribbon synapses of cochlear hair cells: analysis of synaptic vesicle- and synaptic membrane-associated proteins.. Eur J Neruosci.

[76] Chen L, Chetkovich D, Petralia R, Sweeney N, Kawasaki Y (2000). Stargazin regulates synaptic targeting of AMPA receptors by two distinct mechanisms.. Nature.

[77] Tomita S, Byrd R, Rouach N, Bellone C, Venegas A (2007). AMPA receptors and stargazin-like transmembrane AMPA receptor-regulatory proteins mediate hippocampal kainate neurotoxicity.. Proc Natl Acad Sci.

[78] Gregory F, Bryan K, Pangrsic T, Calin-Jageman I, Moser T (2011). Harmonin inhibits presynaptic Ca_V_1.3 Ca^2+^ channels in mouse inner hair cells.. Nat Neurosci.

[79] Boëda B, El-Amraoui A, Bahloul A, Goodyear R, Daviet L (2002). Myosin VIIa, harmonin and cadherin 23, three Usher I gene products that cooperate to shape the sensory hair cell bundle.. EMBO J.

[80] Weil D, El-Amraoui A, Masmoudi S, Mustapha M, Kikkawa Y (2003). Usher syndrome type 1 G (USH1G) is caused by mutations in the gene encoding SANS, a protein that associates with the USH1C protein harmonin.. Hum Mol Genet.

[81] El-Amraoui A, Petit C (2005). Usher I syndrome: unraveling the mechanisms that underlie the cohesion of the growing hair bundle in inner ear sensory cells.. J Cell Sci.

[82] Lillo C, Kitamoto J, Williams DS (2006). Roles and interactions of Usher 1 proteins in the outer retina.. Adv Exp Med Biol.

