# Supplementary figures and images for: Role for a Novel Usher Protein Complex in Hair Cell Synaptic Maturation
Source: PLoS One. 2012 Feb 17;7(2):e30573. doi: 10.1371/journal.pone.0030573 (PMC3281840; doi:10.1371/journal.pone.0030573)

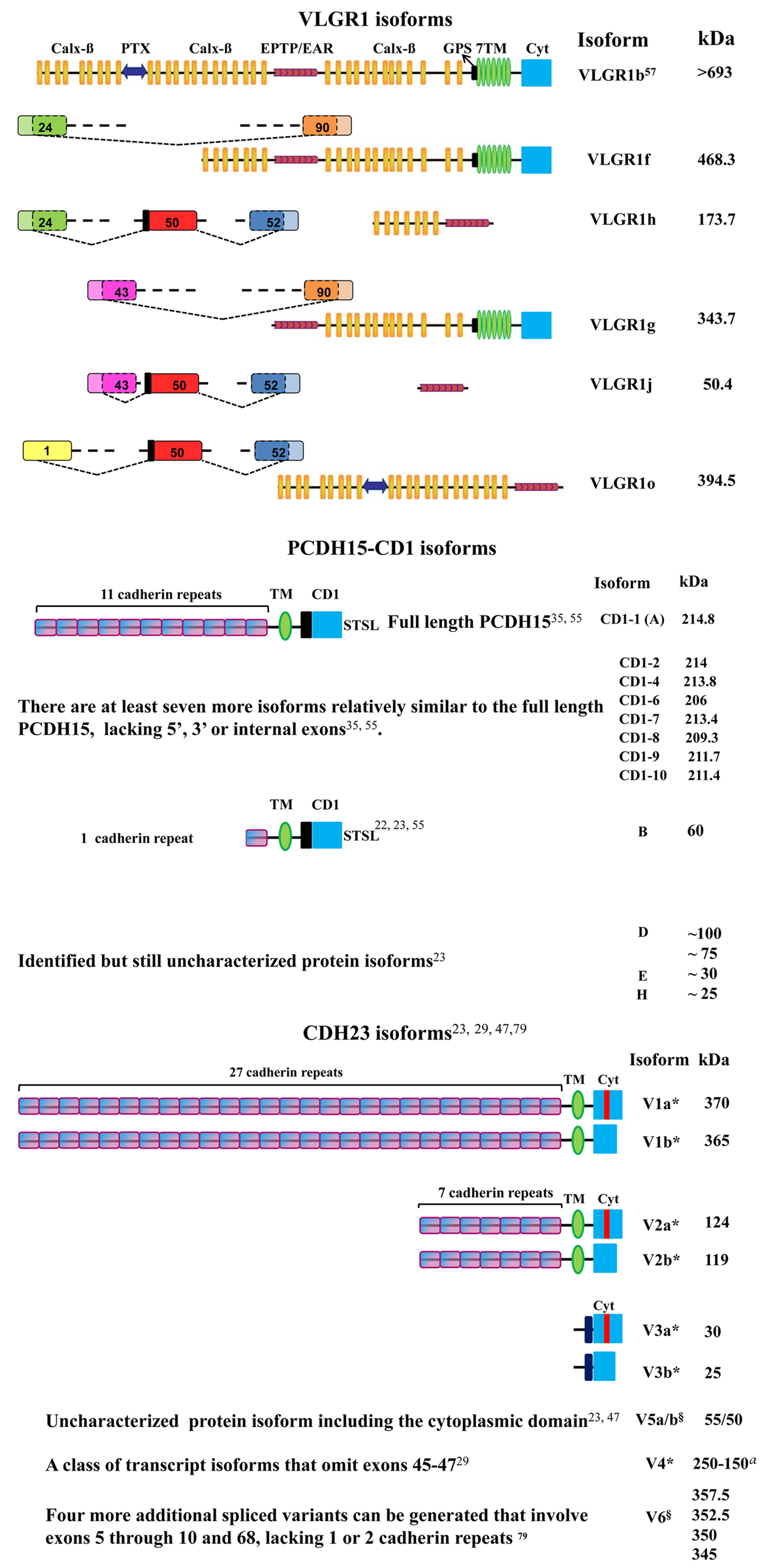

Supplement: Figure S1 — Schematic representation of the Usher isoforms relevant to this work. VLGR1 isoforms: Predicted structure and domains of the full length VLGR1 (Vlgr1b). CalX-β: Ca2+-binding domain of the Na+/Ca2+ exchanger molecule. LamG/TspN/PTX: laminin-G/thrombospondin-N/pentraxin homology domains. EAR/EPTP: epilepsy-associated repeat/epitempin repeat GPS: G-protein-couple receptor proteolytic site TM: transmembrane domain. Cyt: cytoplasmic domain. The new transcripts obtained by RACE are also included (Vlgr1f, Vlgr1g, Vlgr1h, Vlgr1j and Vlgr1o). 5′RACE products: 4.9-kb and 780-bp products comprising exons 24 to 48 and 43 to 48, respectively. These partial 5′ transcripts were combined with the rest of the sequence for Vlgr1b or with the novel 3′RACE product, generating Vlgr1f, Vlgr1h, Vlgr1g and Vlgr1j complete transcripts. 3′RACE product: 800-bp product comprising exons 48 to 52. This partial 3′ transcript was combine with the novel 5′RACE products and with the beginning of the transcript for Vlgr1b, generating Vlgr1h, Vlgr1j and Vlgr1o complete transcripts. The protein domains for each new isoform and their estimated molecular weights are also included. PCDH15 isoforms: The full length PCDH15-CD1 isoform (CD1-1 or isoform A) contains 11 cadherin repeats, a transmembrane domain (TM) and the cytoplasmic domain 1 (CD1) that includes the PDZ binding sequence “STSL”. Several isoforms lacking a small part of the full length PCDH15-CD1 have also been described (isoforms CD1-2 to CD1-10). Isoform B containing only one cadherin repeat and several identified but still uncharacterized isoforms containing part of the extracellular domain (D, G and H). Apparent or estimated molecular weights are included. CDH23 isoforms: The full length CDH23 (V1) contains 27 cadherin repeats, a transmembrane domain (TM) and a cytoplasmic domain (Cyt) with or without the coding sequence present in exon 68 (red box). The CDH23 V2 isoforms only contain 7 cadherin repeats and the CDH23 V3 isoforms are cytosol [file pone.0030573.s001.tif]

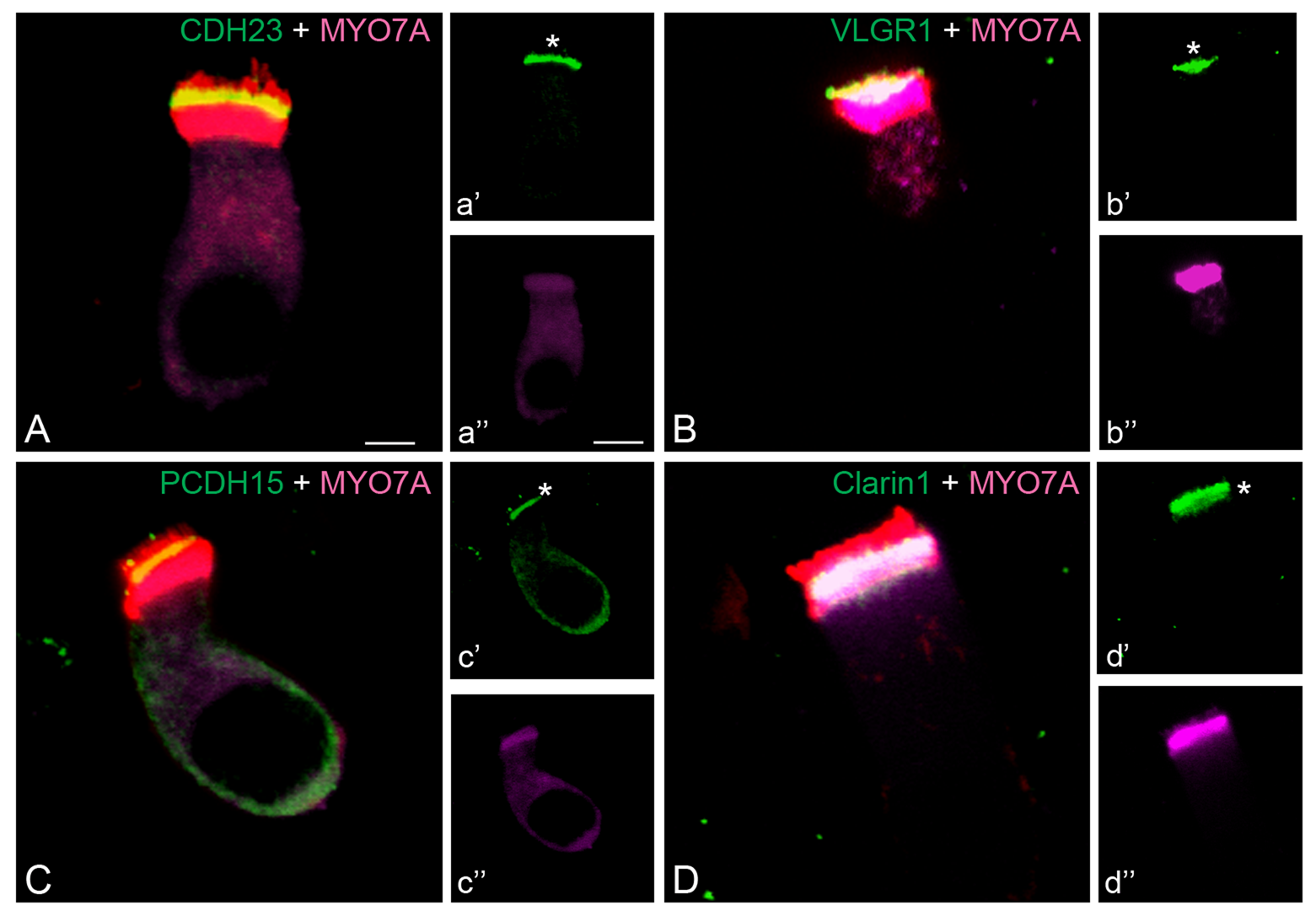

Supplement: Figure S2 — Isolated hair cells from P30 organs of Corti were immunostained for the CDH23 (A, a′, a″), VLGR1 (B, b′, b″), PCDH15 (C, c′, c″) and clarin-1 (D, d′, d″) (green), the hair cell marker myosin7A (magenta) and counter-stained with phalloidin (red). Asterisks: apical staining and co-localization with phalloidin. Scale bar: A–D: 2.5 µm; a′–d″: 5 µm. (TIF) [file pone.0030573.s002.tif]

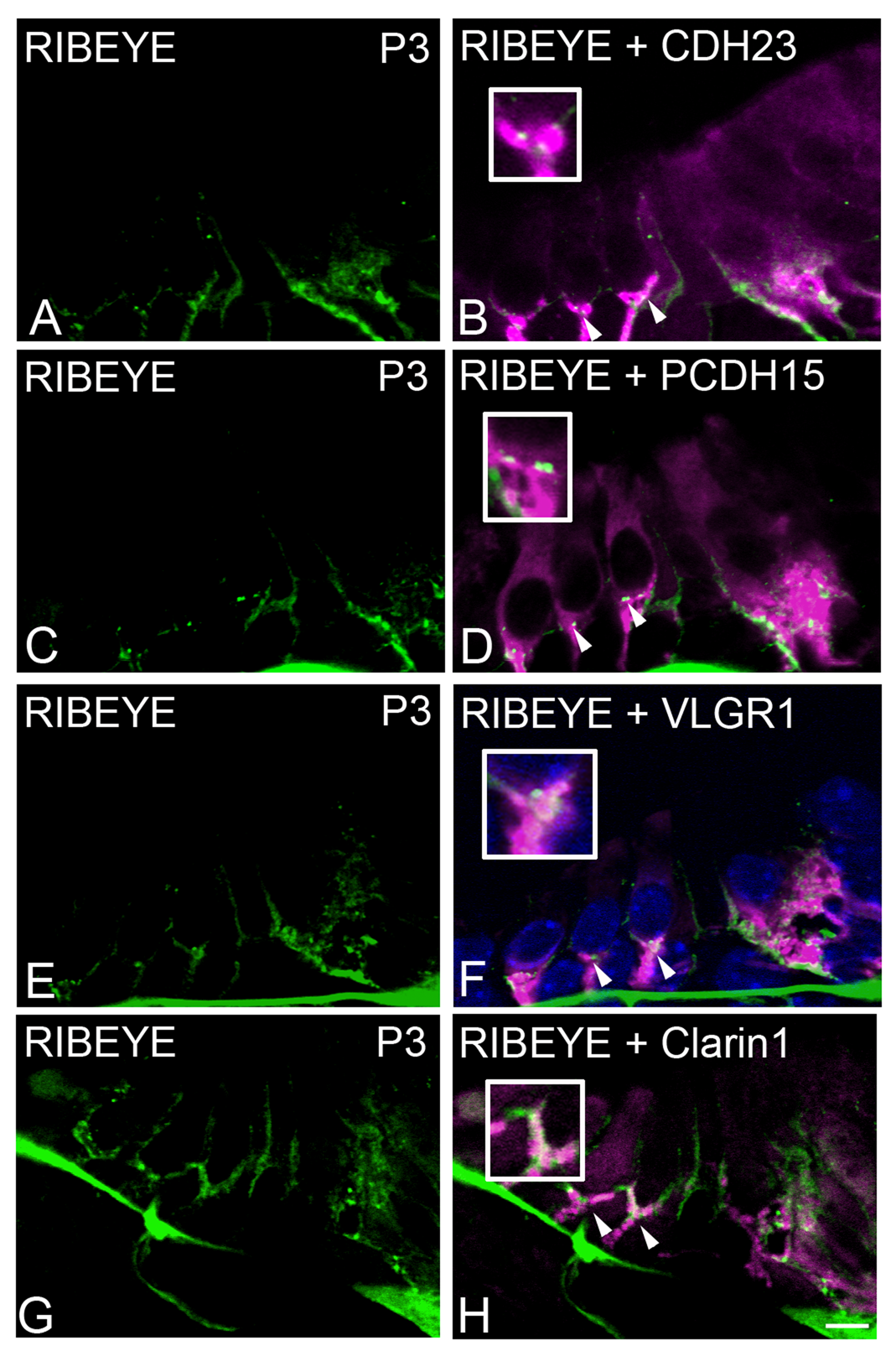

Supplement: Figure S3 — Pre- and post-synaptic expression of CDH23, PCDH15, VLGR1 and clarin-1 in P3 cochleae. Single plane images from P3 cochlea cross-sections immunostained for the Usher proteins (magenta) and the pre-synaptic marker RIBEYE (green). CDH23 (A–B); PCDH15 (C–D); VLGR1 (E–F) and clarin-1 (G–H). Arrowheads denote basal pre-synaptic co-localization in OHCs. Scale bar: 5 µm. (TIF) [file pone.0030573.s003.tif]

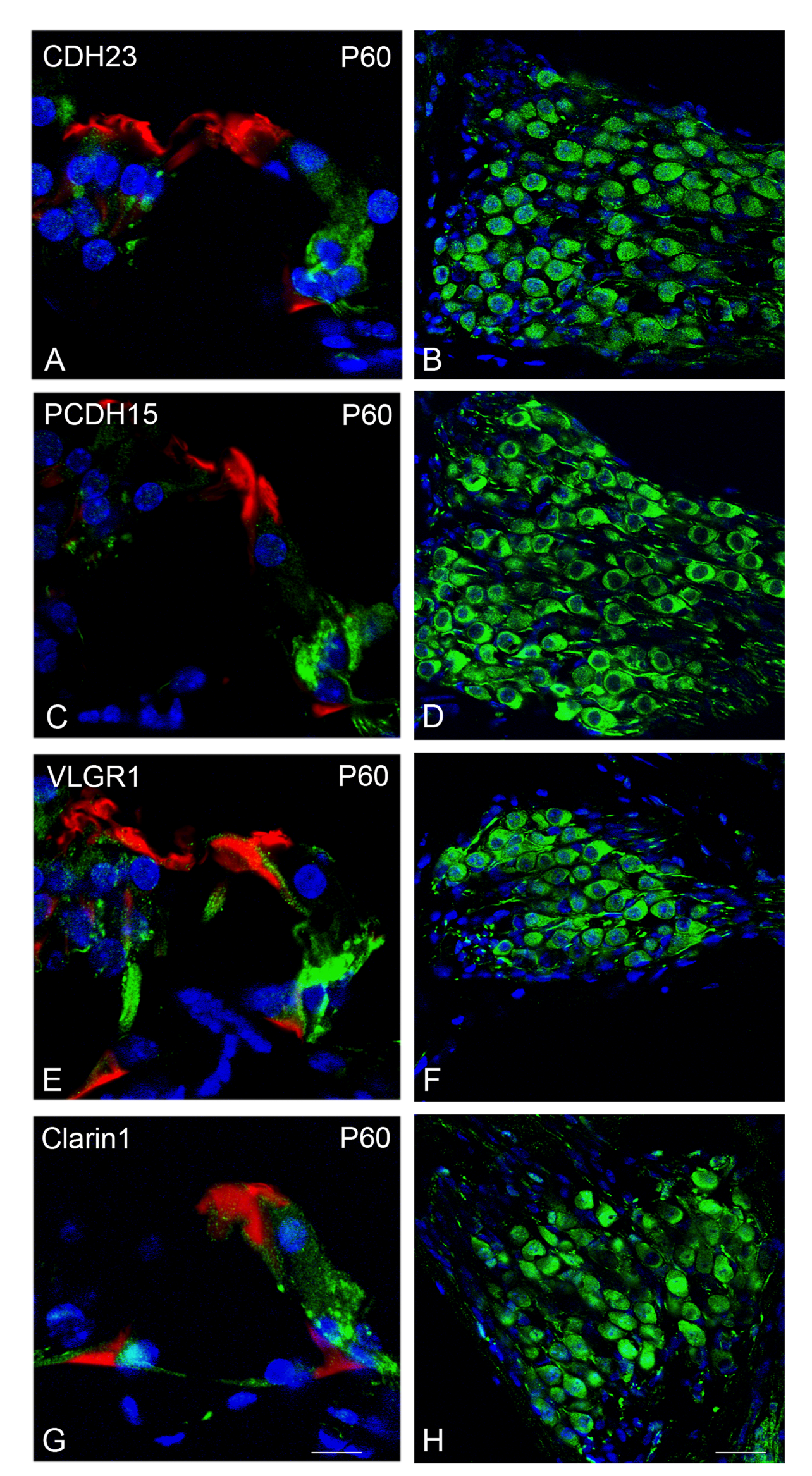

Supplement: Figure S4 — Expression of the Usher proteins in P60 type I afferent neurons. Cochlea cross-sections immunostained for CDH23 (A–B), PCDH15 (C–D), VLGR1 (E–F) and clarin-1 (G–H), showing expression of the Usher proteins in the type I afferent terminals that synapse the IHCs (A, C, D, E, G) and corresponding SGNs (B, D, F, H) . Scale bar: A, C, E, G: 10 µm. B, D, F, H: 25 µm. (TIF) [file pone.0030573.s004.tif]

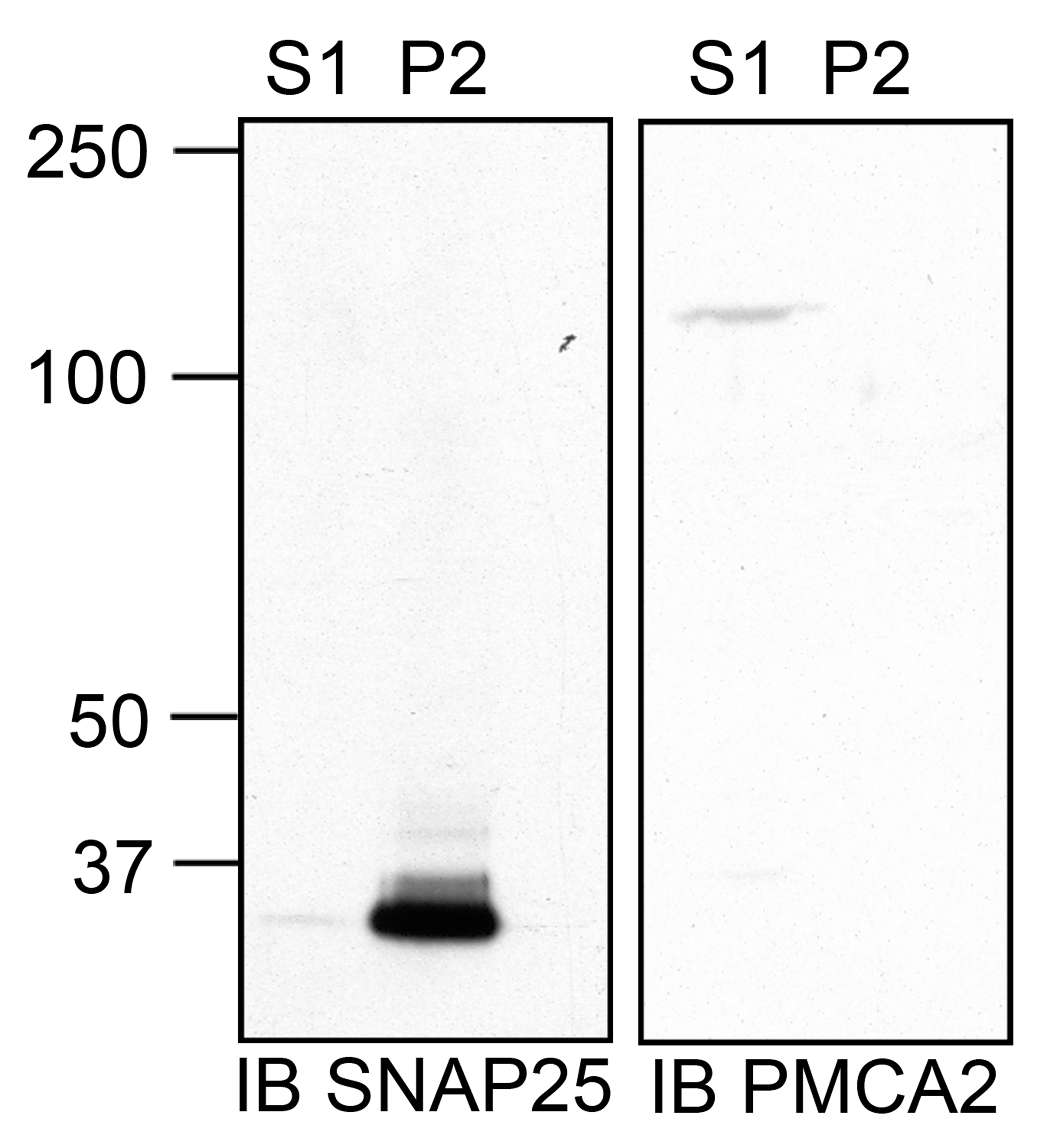

Supplement: Figure S5 — Western blot analysis of synaptosomal preparation from P3 organ of Corti. Left: the synaptic marker SNAP25 was used to demonstrate the presence of the synaptosomal preparation in P2 (see Materials and Methods). Note that the apparent molecular mass of SNAP25 is bigger than expected (25 kDa) as native conditions were used. Right: the apical marker, PMCA2, was used to demonstrate distinct sub-cellular fractionation. PMCA2 is present in S1 and absence from P2 where the crude synaptosomes fractionate. (TIF) [file pone.0030573.s005.tif]

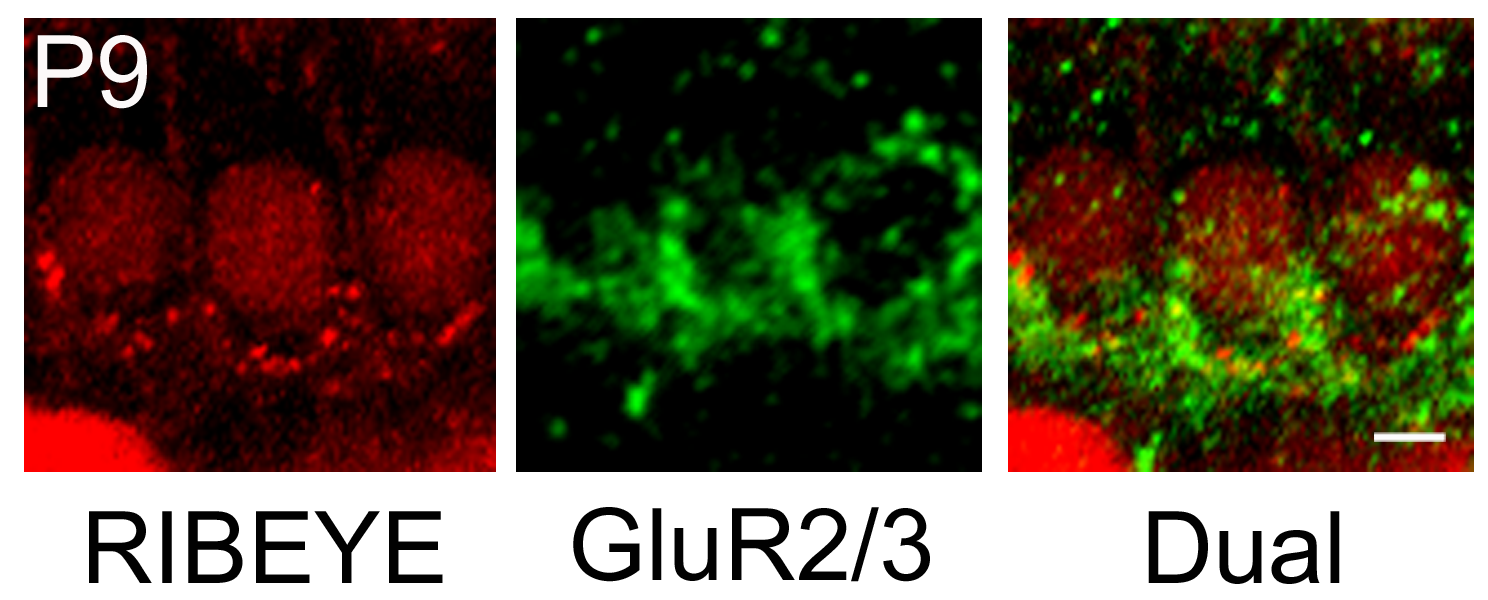

Supplement: Figure S6 — Ames waltzerav3J mouse has immature synaptic contacts. P9 PCDH15 mutant IHC ribbon synapses immunostained for the pre-synaptic marker RIBEYE (red) and the post-synaptic marker GluR2/3 (green). Scale bar: 3 µm. (TIF) [file pone.0030573.s006.tif]

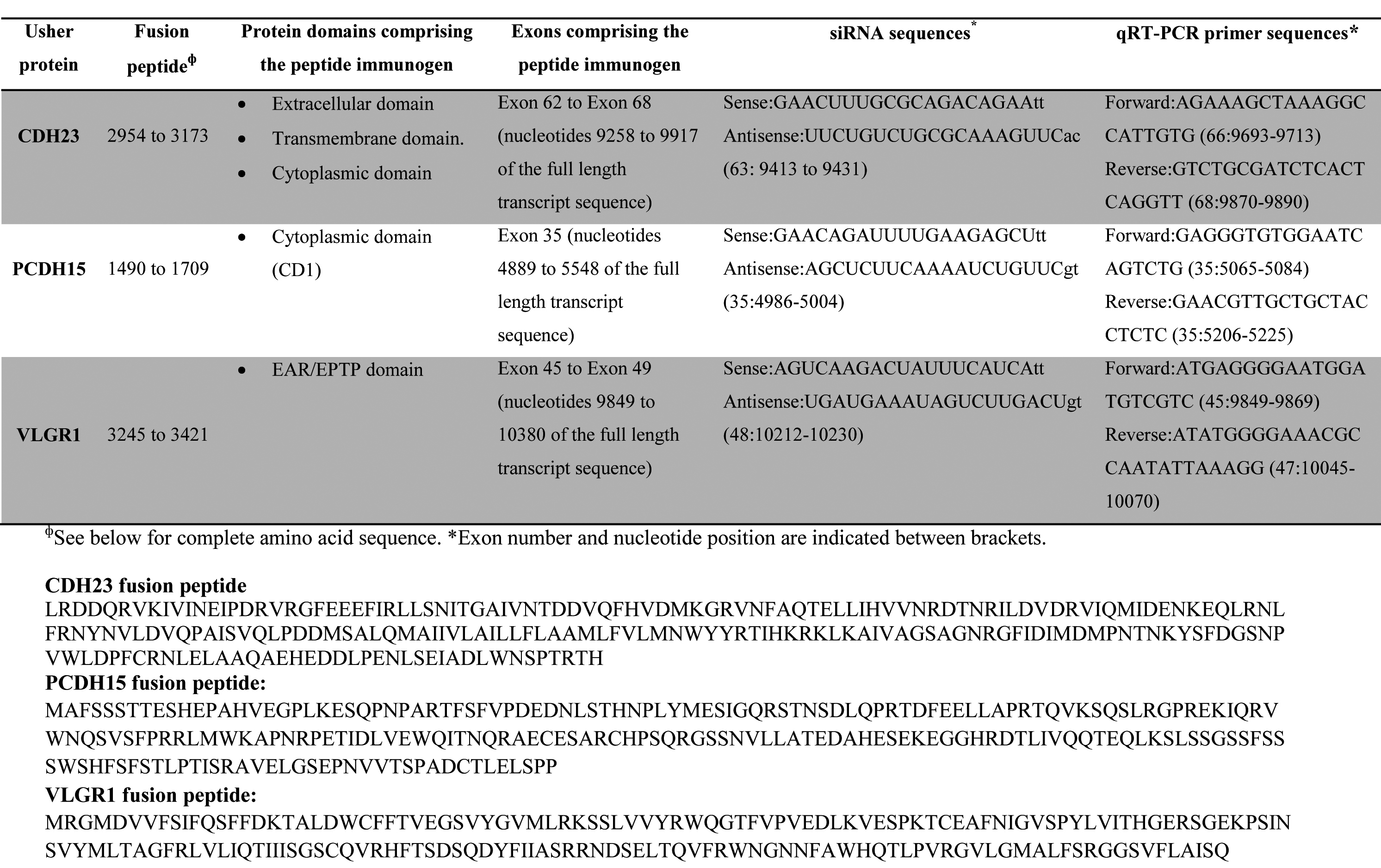

Supplement: Table S1 — Description of the mouse fusion peptides used to generate the corresponding Usher antibodies. siRNA and qRT-PCR primers used for specificity control experiments are also included. Immunogen region for each Usher protein are show below the table. (TIF) [file pone.0030573.s007.tif]
